# The GMC superfamily of oxidoreductases revisited: analysis and evolution of fungal GMC oxidoreductases

**DOI:** 10.1186/s13068-019-1457-0

**Published:** 2019-05-10

**Authors:** Leander Sützl, Gabriel Foley, Elizabeth M J Gillam, Mikael Bodén, Dietmar Haltrich

**Affiliations:** 1 Food Biotechnology Laboratory, Department of Food Science and Technology, BOKU-University of Natural Resources and Life Sciences Vienna, Vienna, Austria; 2 Doctoral Programme BioToP-Biomolecular Technology of Proteins, BOKU-University of Natural Resources and Life Sciences Vienna, Vienna, Austria; 3 School of Chemistry & Molecular Biosciences, The University of Queensland, Brisbane, Australia

**Keywords:** GMC oxidoreductase, CAZy family AA3, Sequence similarity networks, Phylogeny, Evolution of oxidoreductases

## Abstract

**Background:**

The glucose–methanol–choline (GMC) superfamily is a large and functionally diverse family of oxidoreductases that share a common structural fold. Fungal members of this superfamily that are characterised and relevant for lignocellulose degradation include aryl-alcohol oxidoreductase, alcohol oxidase, cellobiose dehydrogenase, glucose oxidase, glucose dehydrogenase, pyranose dehydrogenase, and pyranose oxidase, which together form family AA3 of the auxiliary activities in the CAZy database of carbohydrate-active enzymes. Overall, little is known about the extant sequence space of these GMC oxidoreductases and their phylogenetic relations. Although some individual forms are well characterised, it is still unclear how they compare in respect of the complete enzyme class and, therefore, also how generalizable are their characteristics.

**Results:**

To improve the understanding of the GMC superfamily as a whole, we used sequence similarity networks to cluster large numbers of fungal GMC sequences and annotate them according to functionality. Subsequently, different members of the GMC superfamily were analysed in detail with regard to their sequences and phylogeny. This allowed us to define the currently characterised sequence space and show that complete clades of some enzymes have not been studied in any detail to date. Finally, we interpret our results from an evolutionary perspective, where we could show, for example, that pyranose dehydrogenase evolved from aryl-alcohol oxidoreductase after a change in substrate specificity and that the cytochrome domain of cellobiose dehydrogenase was regularly lost during evolution.

**Conclusions:**

This study offers new insights into the sequence variation and phylogenetic relationships of fungal GMC/AA3 sequences. Certain clades of these GMC enzymes identified in our phylogenetic analyses are completely uncharacterised to date, and might include enzyme activities of varying specificities and/or activities that are hitherto unstudied.

**Electronic supplementary material:**

The online version of this article (10.1186/s13068-019-1457-0) contains supplementary material, which is available to authorized users.

## Background

The glucose–methanol–choline (GMC) superfamily of oxidoreductases was defined by Cavener in 1992 based on sequence similarities of *Drosophila melanogaster* glucose dehydrogenase, *Escherichia coli* choline dehydrogenase, *Aspergillus niger* glucose oxidase, and *Hansenula polymorpha* methanol (alcohol) oxidase [12]. Since then several other enzymes have been identified as members of this superfamily, all of which share a common fold and carry a covalently or non-covalently bound flavin adenine dinucleotide (FAD) cofactor. GMC superfamily members are typically composed of an FAD-binding domain and a substrate-binding domain. The FAD-binding domain contains the strictly conserved Rossmann fold or βαβ mononucleotide-binding motif, while the substrate-binding domain shows more sequence variations depending on the preferred substrates of the respective superfamily member. Commonly known electron donor substrates for GMC oxidoreductases range from various sugars and alcohols to cholesterol and choline. Despite this broad range of chemically diverse substrates, the overall reaction mechanism is similar for these FAD-dependent oxidoreductases. The mechanism can be separated into a reductive (reduction of FAD with concomitant oxidation of the electron donor substrate) and an oxidative half-reaction (re-oxidation of FADH_2_), and relies on a highly conserved catalytic His/His or His/Asn pair in the active site [45, 96, 103]. As the final electron acceptor, GMC oxidoreductases can employ oxygen or alternative electron acceptors such as different quinones, phenol radicals, or metal ions. Varying preferences for these electron acceptors separate GMC enzymes into oxidases (which can utilise O_2_ as electron acceptor) and dehydrogenases (which show negligible or very low reactivity with O_2_).

Glucose–methanol–choline oxidoreductases can be found in yeasts, filamentous fungi, bacteria, and insects [12, 47], and structurally similar but functionally unrelated enzymes also occur in plants [21, 22]. From an applied point of view, GMC oxidoreductases from fungal sources have attracted the most attention with applications of these sugar and alcohol-oxidising enzymes in, e.g., biosensors or the food industry [60, 102]. Recently, possible applications of fungal GMC enzymes were shown in biomass utilisation, as these enzymes can exhibit important auxiliary roles in lignocellulose degradation [8, 62]. Because of this they are summarised in the AA3 family of “Auxiliary Activities” (AA) of the Carbohydrate-Active enZyme (CAZy) database [57], which includes aryl-alcohol oxidoreductase (AAOx; EC 1.1.3.7; AA3_2, and AADH; AA3_2), alcohol oxidase (AOx; EC 1.1.3.13; AA3_3), cellobiose dehydrogenase (CDH; EC 1.1.99.18; AA3_1), glucose oxidase (GOx; EC 1.1.3.4; AA3_2), glucose dehydrogenase (GDH; EC 1.1.5.9; AA3_2), pyranose dehydrogenase (PDH; EC 1.1.99.29; AA3_2), and pyranose oxidase (POx; EC 1.1.3.10; AA3_4). Representatives of all seven of these GMC oxidoreductases have been characterised to date from various fungal sources, crystal structures are available and they were recently reviewed with a focus on their biological functions [88].

In addition to these characterised GMC enzymes, the enormous—and still growing—availability of genomic data for various fungal organisms revealed thousands of putative fungal GMC enzymes, and thus provided us with a recent flood of sequence information. Enzyme superfamilies often contain several thousand sequences, and the investigation of such large data sets, which can harbour significant diversity, is technically demanding [2]. Modern bioinformatics tools offer an option to gain additional information provided by this vast number of fungal GMC sequences. They make it, for example, possible to analyse multiple hundreds or thousands of sequences and thus to greatly enhance evolutionary and comparative studies [10]. Also, novel enzymes and functionalities can be attributed or identified in comprehensive phylogenetic studies. Furthermore, taxonomic distributions and detailed sequence analysis of specific enzymes can give indications about physiological roles of these enzymes. To date, most comparative studies involving sequence and/or structural information are based on a relatively low number of characterised proteins [25, 40, 106], and the vast majority of enzymes within a superfamily remain uncharacterised. Because of this, these comparative studies do not provide information about the position that these well-studied representatives occupy within their respective families. For example, it is not known whether they are a more unique ‘special case’ within their family, setting them apart from other family members, or whether they are close to the core of their family and can, thus, be considered as canonical representatives of their families.

To position characterised sequences in context of other extant sequences of the same enzyme class, a set of sequences covering the entire natural sequence space of this enzyme is needed, and the phylogenetic relationship within this enzyme class needs to be determined. Unfortunately, collecting all currently available sequences for one enzyme proves to be difficult for GMC sequences. In previous studies on members of the fungal GMC superfamily, we found that sequences resulting from genome projects are often annotated simply as ‘GMC oxidoreductase’ without any further indication of their functionalities; in other cases, some were even found to be wrongly annotated [66]. Correct functional classification and annotation of putative sequences were often impossible until now, since no clear similarity cut-offs had been defined to unequivocally group sequences within a distinct class of GMC oxidoreductases. The same problem also affects database searches of GMC enzymes, where search results can quickly reach thousands of entries, and it is not clear up to which similarity cut-off sequences can still be considered to show the same functionality. Grouping such large numbers of diverse sequences in their different classes or clades for functional annotation is not feasible for conventional alignment and tree-building methods. Some sets of sequences are simply too diverse to be aligned, and calculating tree topologies with thousands of sequences is often exceeding a manageable time frame.

To circumvent this problem, we used sequence similarity networks (SSNs) to unambiguously group sequences to one enzymatic function within the GMC oxidoreductases. Such SSNs are known to be well suited for functional clustering of diverse enzyme superfamilies. They provide good visual representations of all sequence relationships in the network, where the similarity cut-off for these relationships can be freely altered to modify and improve the clustering. Compared to calculating multiple sequence alignments and inferring phylogenetic trees, SSNs can handle much larger numbers of sequences in reasonable time [5, 9, 10]. Thus, SSNs are an excellent tool for efficient sampling of the natural sequence space of an enzyme [97].

The aim of this work was to give an extensive overview of the full available sequence space of seven selected GMC oxidoreductases, AAO, AOx, CDH, GOx, GDH, PDH, and POx, as well as to assess their individual phylogenetic relations. This can form the basis for enhanced evolutionary and comparative studies, which can ultimately elucidate how certain enzymatic properties evolved and identify responsible key residues [2]. Our results are finally interpreted from an evolutionary perspective, elucidating the individual histories of some of these GMC enzymes.

## Results

The GMC superfamily is a very large and functionally diverse enzyme superfamily. We, therefore, limited our analysis in this study only to sequences of fungal origin as these enzymes are also of more pronounced applied interest. To ascertain that we study a nonredundant set of all available sequences that can be associated with the GMC superfamily, we first conducted a wide database search and collected all sequences that can be associated with fungal GMC oxidoreductases, and only then narrowed the analysis down to its respective enzymatic functions. BLAST [1] and HMM [75] were used on NCBI and UniProt, respectively, for the database search, which resulted in approximately 10,000 putative fungal GMC sequences. To parse the GMC superfamily into subgroups, we used SSNs. In these networks, the interrelationship between proteins is described as a collection of independent pairwise alignments of their sequences [5]. By selecting suitable stringent threshold values or similarity cut-offs, the sequences break up into distinct subgroups or clusters, in which members of a subgroup/cluster share more similarity among themselves than with members of other subgroups. We then assigned functional information to these individual clusters based on available experimental data and sequence similarity. Single annotated functional clusters were subsequently analysed phylogenetically using MAFFT [48] and PhyML [35] and taxonomic information was retrieved by SeqScrub [29]. Additional sequence analyses beyond phylogenetic relationships further improved the overall view of the studied enzyme clusters.

### Database search and sequence cluster analysis

**Fig. 1:**
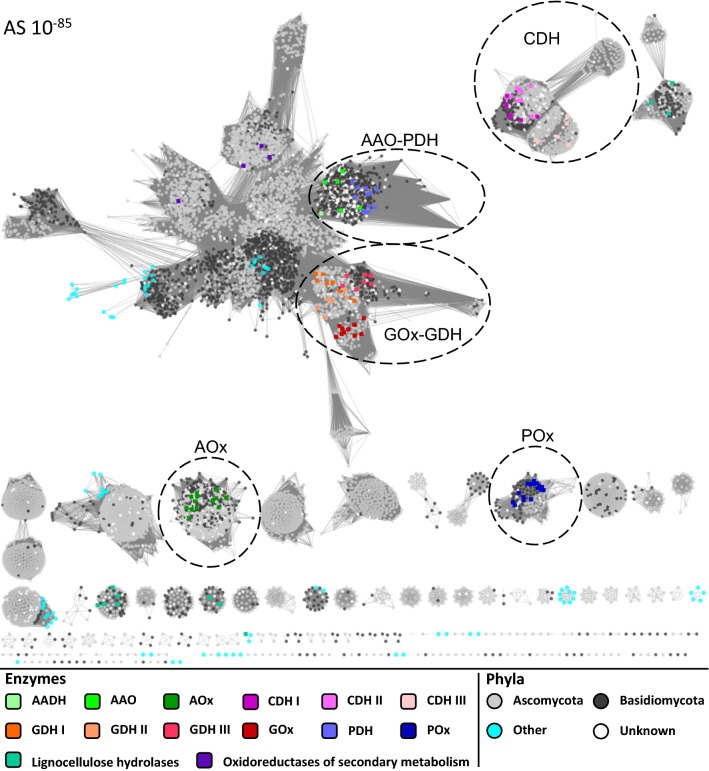
Sequence similarity network at an alignment score cut-off of 10^−85^. The extracted clusters are indicated by dashed circles. Annotated sequences are coloured according to their functionality (‘Enzymes’). All other sequences are coloured according to the fungal phyla they occur in (‘Phyla’)

To discriminate between the different enzyme subfamilies of the GMC superfamily and group them according to their functionalities, we clustered and separated putative sequences based on sequence similarities using SSNs. The SSN was calculated from a total of 9385 unique, nonredundant fungal GMC sequences and visualised in a series of different similarity cut-offs (Fig. 1 and Additional file 1: Figure S1). The similarity cut-off is defined by an alignment score (AS) where a lower AS corresponds to higher similarities of the displayed relations. The sequences used for the calculation originated from two separate database searches based on biochemically characterised GMC sequences, a BLAST search in the nonredundant protein sequences (nr) of NCBI, and a HMM search in TrEMBL and Swiss-Prot of UniProt. In addition, a set of 99 annotated sequences from biochemically or structurally studied enzymes was added and marked for functional annotation of the network. All of these 99 annotated sequences cluster according to their respective functionalities in the network (Fig. 1). We found an additional 15 reviewed Swiss-Prot entries of annotated enzymes in the network. Four of these entries show GMC enzymes that take part in the synthesis of mycotoxins or a quinone epoxide (Versicolorin B synthase, dehydrogenase xptC, dehydrogenase patE, and cyclase atC; termed ‘Oxidoreductases of secondary metabolism’). The remaining 11 Swiss-Prot entries describe enzymes related to lignocellulose degradation (exoglucanase, endoglucanase, endo-1,4-β-xylanase, 4-*O*-methyl-glucuronoyl methylesterase, and 1,4-β-d-glucan cellobiohydrolase; termed ‘Lignocellulose hydrolases’). These latter belong to the glycoside hydrolase and carbohydrate esterase family, and are therefore not part of the GMC oxidoreductase superfamily. The presence of these enzymes in our dataset indicates that the database search was sufficiently extensive to include even several sequences outside of the GMC superfamily. We are, therefore, confident that we covered the vast majority of the currently available sequence space of the fungal GMC superfamily in our analysis.

At the highest and, hence, least specific AS cut-off of 10^−85^ (Fig. 1), certain annotated clusters already appear as disconnected groups of functional similarity (for the annotated GMC enzymes AOx, CDH, and POx), while others are still connected to the main cluster, which at this cut-off includes the GMC enzymes AAO, PDH, GOx, GDH, and oxidoreductases of secondary metabolism. All of the lignocellulose hydrolases (non-GMC) appear in four separate disconnected clusters at this cut-off, away from both the main cluster and the GMC clusters. In addition to these clusters comprising the annotated sequences, a number of areas and separate clusters are discernible in the network. These are completely uncharacterised to date, and it can be expected that they include several new GMC enzymes with potentially novel functionalities.

Considerably, more sequences from Ascomycota than from Basidiomycota (6211 Ascomycota, 2196 Basidiomycota, and 794 sequences of unknown phyla) were found in the SSN, with few sequences from other phyla including Mucoromycota, Chytridiomycota, Microsporidia, or Zoopagomycota. These latter fungal phyla differentiated from Dikarya (Ascomycota and Basidiomycota) around 987 million years ago (MYA) as estimated from TimeTree (http://www.timetree.org/). The fact that some sequences from these phyla are still closely related to sequences from Dikarya indicates a high level of conservation during evolution.

The seven enzyme subfamilies of interest were extracted from the network as part of five separate clusters defined at three different AS cut-offs. The clusters AOx, CDH, and POx already occurred separately at an AS of 10^−85^ (Fig. 1), while the clusters GOx–GDH and AAO–PDH were extracted at an AS of 10^−105^ and 10^−135^, respectively (Additional file 1: Figure S1A and B). These five clusters of seven characterised enzyme subfamilies of the GMC superfamily were then used for more detailed phylogenetic and sequence studies to gain a better understanding of the sequence–structure–function relationship of this enzyme superfamily.

### Phylogenetic and sequence analyses

To make the sets of sequences in the clusters more reliable for phylogenetic analysis, they were further sorted so that they contained only sequences showing intact FAD-binding motifs as well as the catalytic His/His or His/Asn pair. Sequences not showing these features were deleted from the analysis (6–20% of the total sequences for the different enzyme subfamilies were removed that way). Multiple sequence alignments (MSAs) were generated using MAFFT, and were further processed by Gblocks 0.91b to exclude positions with little or no phylogenetic information. Phylogenetic trees were inferred by the maximum likelihood method using PhyML. In the case of the multidomain enzyme CDH, only the dehydrogenase domain (GMC fold) was used for the phylogenetic calculations.

**Fig. 2:**
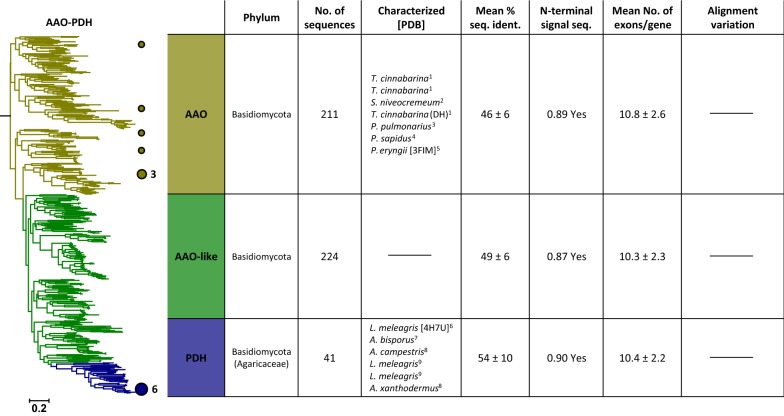
Maximum likelihood tree of the aryl alcohol oxidase–pyranose dehydrogenase (AAO–PDH) cluster. Coloured circles and numbers mark the positions and counts of characterised enzymes in the tree. Right: Table of properties for the three defined clades of the tree. Listed organisms are sorted according to their appearance in the tree (top to bottom). 1—Mathieu et al. [63]; 2—Nagy et al. [68]; 3—Varela et al. [98]; 4—Galperin et al. [30]; 5—Fernandez et al. [25]; 6—Sygmund et al. [89]; 7—Gonaus et al. [33]; 8—Staudigl et al. [87]; 9—Kittl et al. [49]

**Fig. 3:**
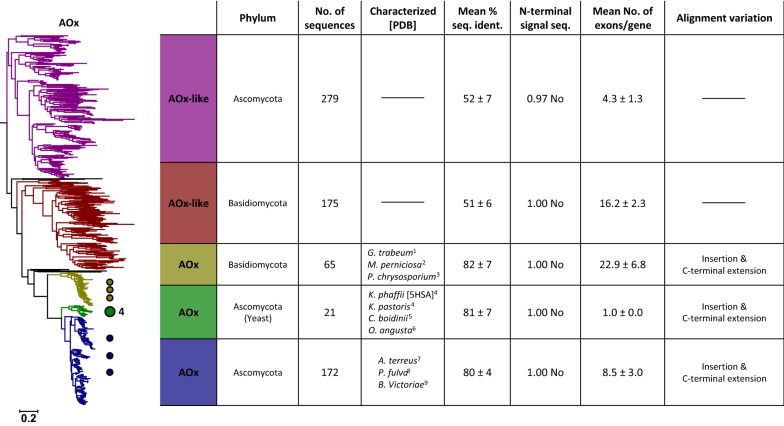
Maximum likelihood tree of the alcohol (methanol) oxidase (AOx) cluster. Coloured circles and numbers mark the positions and counts of characterised enzymes in the tree. Right: Table of properties for the five defined clades of the tree. Listed organisms are sorted according to their appearance in the tree (top to bottom). Black coloured clades were not considered for the analysis. 1—Daniel et al. [15]; 2—de Oliveira et al. [20]; 3—Linke et al. [59]; 4—Cregg et al. [14]; 5—Sakai and Tani [79]; 6—Ledeboer et al. [55]; 7—Chakraborty et al. [13]; 8—Segers et al. [81]; 9—Soldevila and Ghabrial [85]

**Fig. 4:**
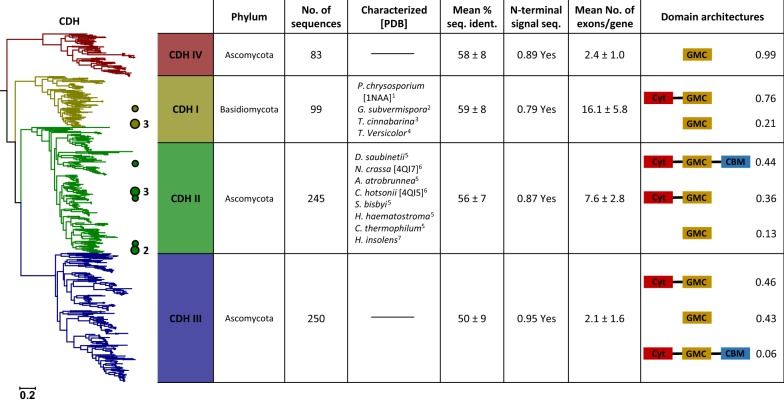
Maximum likelihood tree of the dehydrogenase domains in the cellobiose dehydrogenase (CDH) cluster. Coloured circles and numbers mark the positions and counts of characterised enzymes in the tree. Right: Table of properties for the four defined clades of the tree. Listed organisms are sorted according to their appearance in the tree (top to bottom). 1—Hallberg et al. [39]; 2—Harreither et al. [42]; 3—Bey et al. [7]; 4—Stapleton et al. [86]; 5—Harreither et al. [41]; 6—Tan et al. [95]; 7—Xu et al. [104]

**Fig. 5:**
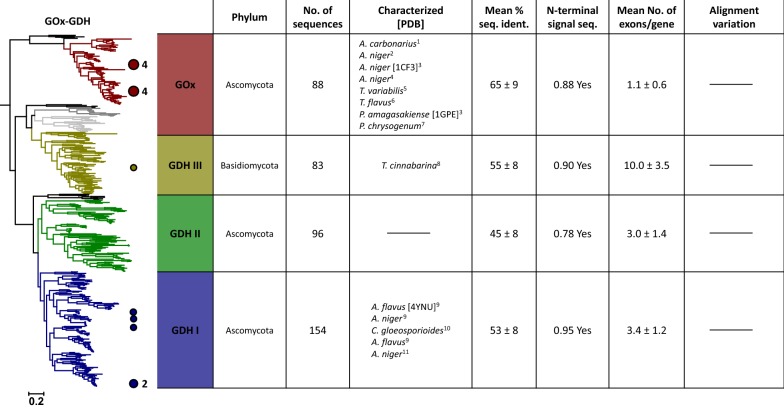
Maximum likelihood tree of the glucose oxidase–glucose dehydrogenase (GOx–GDH) cluster. Coloured circles and numbers mark the positions and counts of characterised enzymes in the tree. Right: Table of properties for the four defined clades of the tree. Listed organisms are sorted according to their appearance in the tree (top to bottom). Black coloured clades were not considered for the analysis. 1—Yang et al. [105]; 2—Hatzinikolaou et al. [44]; 3—Wohlfahrt et al. [101]; 4—Guo et al. [36]; 5—Pulci et al. [77]; 6—Murray et al. [67]; 7—Gao et al. [31]; 8—Piumi et al. [74]; 9—Mori et al. [66]; 10—Sygmund et al. [90]; 11—Sode et al. [84]

**Fig. 6:**
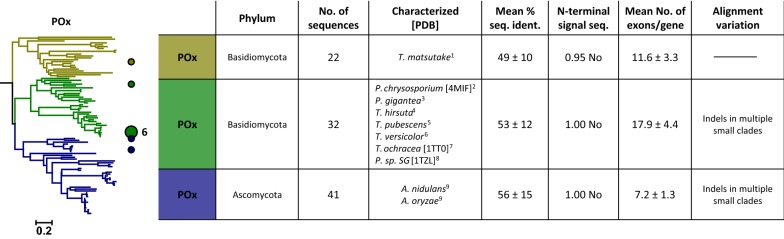
Maximum likelihood tree of the pyranose oxidase (POx) cluster. Coloured circles and numbers mark the positions and counts of characterised enzymes in the tree. Right: Table of properties for the three defined clades of the tree. Listed organisms are sorted according to their appearance in the tree (top to bottom). 1—Takakura and Kuwata [93]; 2—Artolozaga et al. [3]; 3—Danneel et al. [18]; 4—US Patent 6146865, 2000; 5—Maresova et al. [61]; 6—Daniel et al. [16]; 7—Vecerek et al. [99]; 8—Bannwarth et al. [6]; 9—Pisanelli et al. [73]

The five distinct trees that resulted from these phylogenetic analyses were further separated into several clades based on topology, taxonomy, and characterised sequence space. The individual clades were then analysed for additional properties (Figs. 2, 3, 4, 5 and 6). The ‘mean percent sequence identity’ value given here is a measure for the degree of sequence conservation within individual clades. It was calculated from all pairwise sequence identities in the alignment, leaving out gaps, so as to compare the variation within the well-aligned regions of the enzymes only. A higher value for a clade indicates higher evolutionary conservation of its sequences. The fraction of sequences showing an N-terminal signal sequence (and hence an extracellular localisation) was calculated using the SignalP 4.1 server [69]. The number of exons per gene was determined by mapping sequences back to their genome entries and counting the exons making up this sequence. At least 90% (and mostly more than 95%) of the sequences could be mapped back to their genomes for all individual clades except for the POx clade of 32 Basidiomycota sequences, for which we could only map 66% of the sequences to a genome. Most unmappable entries were derived from mRNA data and were not correlated with a genome entry. When comparing these average exon numbers per gene across all clades we observed a general trend that sequences from Basidiomycota contained more exons per gene than those from Ascomycota. For a more detailed study of the origin and taxonomic distribution of sequences forming the different clades, we indicated the positions of GMC sequences according to their clade in a species tree of fungal orders (Additional file 2: Figure S2). In general, individual clades were almost exclusively composed of sequences from only one fungal phylum, Ascomycota or Basidiomycota (see “CDH cluster” for the exception).

### AAO–PDH cluster

The AAO–PDH group was the least defined cluster to be extracted from the SSN, clustering separately from the major cluster of the SSN only at an AS cut-off of 10^−135^. At this threshold value the other GMC clusters had already separated according to their subsequently observed phylogenetic clades. The abbreviation AAO was kept here for historic reasons, but was defined anew as aryl-alcohol oxidoreductases, to include the well-known aryl-alcohol oxidases (abbreviated here as AAOx) as well as the newly identified aryl-alcohol dehydrogenases (AADH) [63]. The taxonomic distribution of the AAO–PDH cluster is limited to Basidiomycota and more specific to Agaricomycetes (Additional file 2: Figure S2A). In contrast to this, sequences from the other clusters are always found in both phyla of Dikarya, Basidiomycota and Ascomycota. We assessed the taxonomic information in the rest of the large cluster containing the AAO–PDH sequences in the SSN with an AS cut-off of 10^−105^ (Additional file 1: Figure S1A) and found that sequences from this cluster occurred across the entire fungal kingdom including various Ascomycota, Basidiomycota, Mucoromycota, and yeasts. A quick phylogenetic analysis of this big cluster using FastTree [76] showed that the most closely related clade to AAO–PDH consists of sequences from Ascomycota and contains the Swiss-Prot-annotated sequence of dehydrogenase xptC, an enzyme in the fungal prenyl xanthone synthesis pathway reducing the aromatic oxo-compound emericellin [80]. Since the most closely related characterised sequence already shows a different functionality to AAO–PDH (although both enzyme families can reduce aromatic compounds like phenols and quinones in the case of AAO–PDH and emericellin in the case of dehydrogenase xptC), we conclude that the AAO–PDH gene does not exist outside of Agaricomycetes. To date, it is unclear why this gene has such a limited taxonomic distribution.

The detailed phylogeny and sequence analysis of the AAO–PDH cluster (Fig. 2) indicate that the cluster is rather uniform (see also Additional file 3: Figure S3A). It does not show significant differences in cellular localisation (signal sequence), number of exons, or alignment structure among the phylogenetic clades, and only the level of sequence conservation (mean percent sequence identity) is somewhat higher for PDH (54%) than for the other two clades (46% and 49%). It was unexpected to see PDH so closely related to AAO, since PDH oxidises carbohydrate substrates more similar to substrates from GOx, GDH, or POx rather than aromatic alcohols, the preferred electron donor substrate for AAO. The AAO clade shown in Fig. 2 also contains the sequence of the aryl-alcohol dehydrogenase (AADH) from *Pycnoporus cinnabarinus*, which is positioned among aryl-alcohol oxidases (AAOx). Aryl-alcohol oxidases and dehydrogenases, thus, do not appear in separate parts of the tree or in separate clades, indicating that only subtle amino acid changes are responsible for the shift of oxygen specificity in this enzyme. All characterised sequences of PDH are found on the opposing end of AAO in the tree, and strictly occur only in the family of Agaricaceae. Given such a narrow taxonomic distribution and little phylogenetic distance to AAO, we can assume that PDH evolved only recently from AAO after a change in substrate specificity in Agaricaceae. Whether this change in functionality leading to the split of these enzymes happened gradually or was a sudden event is still uncertain, since the sequence space between these two clades is completely unexplored to date. A comparison of active site residues may give a first idea of the evolutionary history though. In PDH from *Leucoagaricus meleagris* (*Lm*PDH; PDB 4H7U), four residues show interactions with the sugar substrate, the catalytic H512/H556 pair as well as Q392 and Y510 [96]. In AAO from *Pleurotus eryngii* (*Pe*AAO; PDB 3FIM), a hydrophobic bottleneck is formed by Y92, F397 and F501, regulating substrate access from the solvent to the active site [25]. The only residue found to be involved in aryl-alcohol substrate binding other than the catalytic H502/H546 pair is again Y92 [26, 27]. We compared sequence logos of all of these active-site residues for the three different clades, AAO, AAO-like, and PDH (Additional file 4: Figure S4), with the exception of F397 from *Pe*AAO as this position was not well enough aligned throughout all three clades. The catalytic His/His pair is well conserved in all three clades, with a small fraction of sequences showing a His/Asn pair. While Q392 from *Lm*PDH is not strictly conserved, most PDH sequences show a polar residue at the corresponding position. In contrast, AAO and AAO-like show mostly aliphatic residues. At position Y510 of *Lm*PDH, the aromatic property of this residue is mostly conserved in PDH, while AAO and AAO-like sequences show aliphatic or polar residues. In the *Lm*PDH crystal structure (PDB 4H7U), this aromatic residue is positioned to allow a hydrophobic stacking interaction with the sugar substrate. Such CH/π bonds are commonly observed in sugar-binding proteins [4, 39]. The aromatic residues forming the hydrophobic bottleneck in *Pe*AAO (Y92 and F501) are mostly conserved in the AAO clade but absent in PDH. At these positions, AAO-like sequences show a transitional architecture where aromatic residues are still present but occur together with less bulky aliphatic residues. We conclude that a possible starting point for the shift in substrate specificity might have been the loss of the narrow hydrophobic channel, which opened up the active site for other substrates such as sugars. Whether enzymes from the AAO-like clade actually have an extended substrate specificity compared to AAO still needs to be determined experimentally. Another feature that is unique for PDH compared to AAO is a covalent linkage between the FAD cofactor and the polypeptide chain. The His residue responsible for forming this covalent linkage in *Lm*PDH (His103) is conserved in 93% of all sequences in the PDH subgroup (Additional file 4: Figure S4). The same position in the AAO and AAO-like clades is occupied by hydrophobic residues (mainly Ile or Val) in 87% and 92% of the sequences, respectively. The development of a covalently linked FAD might have additionally affected the separation of PDH from AAO by altering the redox potential of FAD and thereby changing the active sites’ reactivity [34, 46].

### AOx cluster

Phylogenetic analysis of the AOx cluster shows a split into five clades (Fig. 3). Three clades include sequences of several well-studied AOx members and group according to taxonomy in Basidiomycota, Ascomycota, and Saccharomycetes (yeast), a division of Ascomycota. The other two clades observed in the AOx cluster are completely uncharacterised to date, but appear closely related to AOx and were, therefore, named AOx-like. Similar to AOx, they appear in Ascomycota as well as in Basidiomycota and show a very similar taxonomic distribution pattern (see Additional file 2: Figure S2B). Apart from their close phylogenetic relationship and similar taxonomic distribution, AOx-like sequences show several distinct differences to AOx. Their mean sequence identity is ~ 51% compared to ~ 69% across all three AOx clades, pointing towards stronger evolutionary constraints in AOx and therefore a higher degree of conservation. This difference can be easily visualised by the alignment overview (Additional file 3: Figure S3B) or the shorter phylogenetic distance within the AOx clades (Fig. 3). Other differences between AOx-like and AOx sequences that can be seen in the alignment overview (Additional file 3: Figure S3B) are related to a relatively long insertion close to the C-terminus and a C-terminal extension, both forming extended loop structures, observed for all AOx sequences. Both of these regions were described as relevant for oligomerisation of the homooctameric AOx from *Komagataella phaffii* (formerly *Pichia pastoris*) (*Kp*AOx; PDB 5HSA) based on structural studies [50]. The positioning of both of these regions at the interface of individual subunits is highlighted in Additional file 5: Figure S5. The *Kp*AOx octamer can be described more precisely as a dimer of tetramers, with the C-terminal extensions interlinking all units of one tetramer (Additional file 5: Figure S5A), and the insertion of one subunit interacting with two other subunits from both tetramers (Additional file 5: Figure S5B). Since these two structural features important for oligomerisation are completely missing from AOx-like enzymes, they are likely to display a different degree of oligomerisation.

The C-terminus may also play an important role in cellular localisation of AOx. Yeast AOx has been shown to be peroxisomally localised and to contain a conserved C-terminal peroxisomal targeting signal (PTS) encoded by the last three residues of the polypeptide chain [70]. This PTS was also found for some other Ascomycota sequences but not for sequences of Basidiomycota, which in contrast were shown to associate with the hyphal periplasmic space and cell walls, as well as with extracellular slime [15, 20]. To see if these findings also hold true for the sequences studied here, we created sequence logos for the last ten residues of all 712 sequences of the five AOx subgroups (Additional file 6: Figure S6). We found the PTS to be well conserved only in AOx of Ascomycota (including yeast), showing the consensus sequence ARF in Saccharomycetes and SRL in filamentous ascomycetes. The subgroup of Basidiomycota AOx contained a partly conserved Arg at the last position, while both AOx-like clades were missing a conserved C-terminus entirely. Since all sequences of the AOx cluster also failed to show any N-terminal signal sequence, the unknown AOx-like sequences are predicted to be cytosolic enzymes.

### CDH cluster

Cellobiose dehydrogenase is a multidomain protein composed of a GMC dehydrogenase (DH) domain, a cytochrome (Cyt) domain, and in some instances a carbohydrate-binding module (CBM). The phylogenetic analysis of the CDH cluster was based on alignment positions from the DH domain only. In addition to the three clades CDH I, CDH II, and CDH III, which had already been described in literature [41, 107, 108], we observed another clade of CDH-like sequences in the cluster, termed CDH IV. CDH IV only occurs in ascomycetes and is evolutionarily the most distant clade of CDH (Fig. 4). Sequences in this clade strictly show the DH domain only and not the Cyt domain, which is mostly present in class I–III, but can be missing in these classes as well (Fig. 4 and Additional file 3: Figure S3C), e.g., 43% of all sequences of the CDH III clade do not contain a Cyt domain. This regular occurrence of clades lacking a Cyt domain across the entire CDH cluster suggests that the Cyt domain of CDH was lost during evolution and, thus, that the Cyt domain is not strictly necessary for all of the physiological functions of CDH in certain fungi. The presence of the Cyt domain was thought necessary for archetypal CDH sequences, and it is conceivable that sequences lacking the Cyt domain have, therefore, been overlooked as cellobiose dehydrogenases before. However, in vitro truncated DH domains of CDH were previously used for biochemical studies confirming enzymatic activity for the FAD-containing DH domain alone [51, 91, 95], and therefore, we can assume that CDH sequences lacking a Cyt domain will show activity. Another domain known to occur in CDH is a C-terminal CBM, which is mainly found in class CDH II and divides it further into CDH IIA and CDH IIB depending on the presence or absence of a CBM, respectively [41]. Our analysis showed that a CBM may also occur in CDH III, but only in a small subset of sequences (Fig. 4). Many sequences in class CDH III (137 sequences, corresponding to 55%), however, show an additional C-terminal extension (Additional file 3: Figure S3C), which does not match any known domain sequence and, hence, the function of which remains unknown. It should be noted that no class III or class IV CDH has been characterised biochemically to date.

Contrary to previously published topologies [41, 108], we found that the two Ascomycota clades CDH II and CDH III are more closely related to each other than are CDH I and CDH II (Fig. 4). This general topology is also supported by a phylogenetic analysis based only on the Cyt domains found in the CDH cluster (Additional file 7: Figure S7). The fact that these two independent phylogenetic trees show the same general topology indicates that both domains, DH and Cyt, shared the same evolutionary history and that there was most probably one historic fusion event of a GMC DH domain and a Cyt domain, which created an ancestral CDH prior to the evolution of the three clades CDH I, CDH II, and CDH III. Since the CDH IV clade does not show a Cyt domain, it may have been the first clade to have lost the Cyt domain again very early in its evolution, or is a direct descendent of the ancestral GMC enzyme that originally fused with a Cyt domain.

Interestingly, seven CDH sequences from Basidiomycota (Order: Agaricales) were found to cluster within the clades CDH II or CDH III, which otherwise are exclusively composed of sequences from Ascomycota, suggesting a horizontal gene transfer (HGT) from Ascomycota to Basidiomycota (see Additional file 2: Figure S2C). Six of these sequences that cluster in clade CDH III (A0A0D7AQ43, A0A0D7AEU6, A0A0D7AEP1, PBK68988, PBK99967, SJL13846) are from *Fistulina* (three sequences) and *Armillaria* (three sequences), and one CDH II sequence (A0A219WGI5) is from *Termitomyces clypeatus* [78]. Typically, CDH sequences from Basidiomycota lack a C-terminal domain or extension (Additional file 3: Figure S3C), yet two of these seven Basidiomycota sequences, CDH III A0A0D7AEP1 from *Fistulina hepatica* and CDH II A0A219WGI5 from *T. clypeatus*, show a CDH III typical C-terminal extension and an intact CBM domain, respectively. Additionally, they show complete Cyt domains, which also cluster with clade CDH II and CDH III, respectively, in the phylogenetic tree of only Cyt domains. These sequence characteristics are not present in the five remaining Basidomycota sequences. The presence or absence of such Ascomycota-specific features indicate different time points of HGT among these Basidiomycota CDHs, with *F. hepatica* and *T. clypeatus* probably being the most recent.

### GOx–GDH cluster

The cluster of GOx–GDH separates into four main clades: GOx, GDH I, GDH II, and GDH III, as we reported recently [88]. Now, we identified several additional minor clades, but we omitted these from the GOx/GDH classification and the analysis given in Fig. 5 because of their small numbers of sequences (11, 21, and 8 sequences for the minor clades marked in dark-grey, light-grey, and with dashed lines in the phylogenetic tree shown in Fig. 5). Clade GDH II is completely uncharacterised to date and only one sequence was expressed and studied from clade GDH III. All other so-far characterised enzymes belong to the clades of GOx and GDH I. The GOx clade appears completely separated from all clades containing glucose dehydrogenases and is not associated with GDH III as previously reported [88]. It should be mentioned that it is particularly difficult to correctly place the GOx clade within the GOx–GDH topology, since this clade shows a very long branch and therefore, variations in different topologies can be expected. Compared to the taxonomically well-distributed GDH clades, GOx is mainly found in Eurotiales, and in lower numbers also in Helotiales and Hypocreales (see Additional file 2: Figure S2D). This limited taxonomic distribution can be either a result of an extensive gene loss among fungal organisms or a specialisation taking place only in certain orders of fungal organisms. The GOx clade also shows the highest mean value of percent sequence identity in the cluster. Furthermore, in contrast to GDH, most GOx sequences (92%) show only one exon per gene, which we only observed for the AOx gene of yeasts otherwise. Interestingly, one of the minor clades of Ascomycota sequences (Fig. 5 dark-grey lines) shares this low number of exons. This clade of 11 sequences is closely associated with the Basidiomycota sequences of clade GDH III, similar to what has been observed for GOx in alternative topologies. Additionally, the taxonomic distribution of this small clade is limited to three orders of Pezizomycotina (Dothideales, Capnodiales, and Xylariales) and does not overlap with GOx (see Additional file 2: Figure S2D). We conclude that this minor clade probably evolved together with GOx but did not conserve as well in fungal genomes, with currently only 11 discovered sequences.

The most closely related clade to the minor one described above is another small clade (Fig. 5 light-grey lines) of 21 Basidiomycota sequences, only occurring in Ustilaginomycotina and also closely related to GDH III. As shown by the taxonomic distribution (Additional file 2: Figure S2D) no overlap of the origin of these sequences with GDH III occurs, indicating that they are the Ustilaginomycotina equivalent of GDH III. In contrast to GDH III though, this minor clade shows an insertion for most sequences (Additional file 3: Figure S3D) at a position that corresponds to the same location in the structure as the above-mentioned insertion of AOx (see “AOx cluster”) and the head domain of POx [40], both hypothesised to play a role in oligomerisation. Another minor clade displaying an interesting feature in the alignment was identified outside of the two Ascomycota clades GDH I and GDH II (Fig. 5 black dashed lines). The sequences’ taxonomic distribution is limited to Dothideomyceta (Capnodiales and Botryosphaeriales) and they show a well-conserved deletion of a loop of ~ 17 amino acids close to the N-terminus (Additional file 3: Figure S3D). Structural studies of the closely related GDH from *Aspergillus flavus Af*GDH (*PDB* 4YNU) showed that this loop contains Y53, one of the residues responsible for the high preference of this enzyme for glucose over maltose [28]. The structure of *Af*GDH also shows that this loop partly covers the active site entrance, leaving the FAD much more exposed when absent, as in the sequences of this minor clade. Both the lack of the discriminating Y53 and the open active site indicate that members of this minor clade may display a more relaxed substrate specificity compared to GOx and GDH.

### POx cluster

The POx cluster was by far the smallest cluster with a total of 95 sequences, which is about one-fifth of the number of sequences of the second smallest cluster. Despite this low number of sequences, POx displays a widespread taxonomic distribution comparable to the other clusters in this study. This discrepancy may be explained by a significant POx gene loss that apparently happened in many individual fungi. POx was hypothesised to be introduced into fungi via HGT from bacteria [49] and thus, the functions of POx might have been redundant in a number of fungal organisms leading to its subsequent loss [73].

Our phylogenetic analysis of POx shows three main clades, two containing sequences from Basidiomycota and one from Ascomycota (Fig. 6). Note that the two Basidiomycota clades do not cluster together. POx members from all three clades have already been characterised with a strong focus on sequences from Polyporales (Basidiomycota). The second Basidiomycota clade, containing only one characterised sequence, appears as the most distant clade of the three. Interestingly, we found that this clade contains a sequence of Mucoromycotina, a subphylum of fungi that separated from Dikarya (Ascomycota and Basidiomycota) over 900 MYA ago (http://www.timetree.org/). None of the three clades shows an N-terminal signal sequence or any other conserved motif for cellular localisation, despite POx having been shown to be an extracellular enzyme found to be associated with membrane-bound vesicles or other membrane structures [17]. This localisation, as well as the absence of any known signal sequence, is similar to the AOx sequences of Basidiomycota (see “AOx cluster”).

As visualised by the alignment overview (Additional file 3: Figure S3E), POx shows the highest number of insertions and deletions of all clusters and can, therefore, also be expected to show the most variations in its structures. This is also corroborated when comparing the structures of POx from *Trametes ochracea* and *Phanerochaete chrysosporium* ([40], 1TT0; [43], 4MIF). These show an unusual degree of structural differences for the otherwise well-conserved overall conformation of the GMC superfamily. Many differences that are obvious from the sequence alignment are each restricted to a relatively small number of representatives. Although the detailed evolutionary history of POx is still unknown, it clearly is the most ‘unusual’ or ‘atypical’ fungal GMC superfamily member with a high level of sequence variation. This may be attributed to a bacterial origin of the POx gene. A functional POx from bacterial source was only recently described [64], and a database search for analogues of the POx gene that was extended to bacterial sequences showed that these genes can indeed be found in a number of bacterial genomes (we identified 278 POx genes in bacterial genomes in total), consistent with the possibility of a transfer from bacteria to fungi.

## Discussion

The dataset used in this work was derived from two databases, and two different search algorithms were employed to include as much sequence information as possible. The available data on fungal sequences from such databases are biased to a certain extent because of an uneven coverage of sequence data for different fungal classes or even phyla, with certain fungal phyla underrepresented while multiple genomes of a single fungal species might be available. To counter the bias in sequence numbers from more frequently sequenced species as well as to remove redundant sequences that are the result of two independent database searches, we deleted all but one sequence from groups showing ≥ 99% sequence identity. As a result, our dataset does not necessarily contain all GMC sequences of a single organism and therefore cannot be used for, e.g., studies on the multigenicity of GMC enzymes in one specific fungus. The dataset rather represents an overview of the currently known sequence space of fungal GMC oxidoreductases and we are confident that the vast majority of this sequence space is covered in this study.

Phylogenetic studies in fungi are complicated by the fact that fungal genomes are highly variable [65]. Gene losses, gene and whole genome duplications, transposable elements, as well as high mutation rates for genes reacting to environmental changes can lead to high sequence variations even between closely related fungal species [23, 65, 92]. As a result, when comparing previous phylogenetic analyses of GMC sequences [26, 27, 41, 90, 107], it can be seen that the assessed topologies for the same enzymes vary strongly between different studies. While the identification of individual clades and their respective compositions are mostly stable and in agreement among different studies, the phylogenetic relationships between them are strongly dependent on the sequences selected for their construction, the alignment, and the tree-building algorithm. This unstable relationship is independent of the node support of a specific tree, which only evaluates how well a tree represents a specific dataset and cannot indicate if a tree is actually correct [71]. An unstable relationship can be observed both between individual enzyme families (e.g., AOx, CDH, GOx) as well as between classes and clades of these enzyme families (e.g., CDH I, CDH II, and CDH III). In this current work, we aimed to include as much phylogenetic information as possible in the analysis, while at the same time reducing the signal noise from highly variable regions. Nevertheless, we found that topologies for the clusters AAO–PDH, CDH, and GOx–GDH were more easily affected by the sequence selection and alignment algorithm while topologies for the clusters AOx and POx were more stable. Generally, as a result of the substantially larger variety of sequences included in the calculations, the phylogenetic trees obtained in this study can be expected to be more reliable, and reflect the phylogenetic relationship among individual clades more accurately, than previous topologies.

The level of sequence conservation within a clade was measured as the mean percent sequence identity within that clade and we compared these values independent of the number of sequences making up a clade. We argue that this is a valid comparison since the extant level of sequence conservation for a gene is not determined by its possibilities of free change (which is dependent of the number of sequences), but rather by selection pressure implied through biological function. This is also what we observed in our analysis. Highly conserved sequences, such as those of AOx, show a higher mean percent sequence identity independent of the number of sequences in the clade and smaller clades can show similar or even lower levels of conservation compared to larger clades from the same cluster, as observed in the clusters of CDH, GOx–GDH, or POx. The calculated mean percent sequence identity ranged from 45% for GDH II up to 82% for AOx, with the highest values of sequence conservation found for enzymes that showed a more specific substrate range, such as AOx and GOx, and as such are under a more restrictive selection pressure than enzymes with a broad substrate range. It should be noted that especially those clades that do not contain biochemically characterised members often show lower levels of conservation. Although all sequences in the clusters were screened to contain all necessary motifs and active site residues required to be theoretically active, we cannot rule out the possibility that these clades contain some pseudogenes, and therefore display a higher level of sequence variation than functional forms of the gene.

Looking specifically at the conservation of oxygen reactivity within the clusters of fungal GMC oxidoreductases, we could find some variation for different groups of enzymes. For AOx as well as for GOx, all characterised sequences within a clade show a highly conserved oxygen reactivity. Additionally for GOx, this clade is clearly distinct from closely related dehydrogenases. For AAO on the other hand, oxidases (AAOx) and dehydrogenases (AADH) occur dispersed in the same clade, showing that there is no stringent selection towards oxygen reactivity for this enzyme. For POx, although sequences lacking oxygen reactivity have not been reported to date, the dehydrogenase activity might really be the biologically more relevant function [72]. Not all oxidases might, therefore, have evolved strictly as producers of hydrogen peroxide. Instead, it may be that for some enzymes, oxygen reactivity only evolved as a side reaction, while their dehydrogenase function is the biologically more relevant one. Such unspecific oxygen reactivities further complicate studies aiming to discover the principle of oxygen reactivity in flavoenzymes.

For some fungal enzymes, evolutionary histories are not easy to trace due to the high variability and adaptability of fungal genomes. This also goes for POx, an enzyme that has been hypothesised to have been introduced into fungi through horizontal gene transfer (HGT) from bacteria. The distribution of POx in the fungal kingdom is somewhat peculiar when compared to the other enzyme members of the GMC superfamily. POx is rarely found in two closely related fungal species [73] and is generally found only in few species but still throughout most of the fungal kingdom. If one ancient HGT was the origin of fungal POx, then that HGT must have happened very early in fungal evolution followed by a massive gene loss in most fungi. A similar taxonomic distribution pattern and explanation was recently reported for vanillyl alcohol oxidases, a fungal flavoenzyme hypothesised to originate from a HGT from bacteria [37]. An alternative explanation for these fragmented taxonomic distributions in the species tree would be for HGTs to happen much more frequently than assumed until now. To that end either multiple HGT events from bacteria to fungi or HGTs between fungi would be conceivable. Indications for such regular HGTs between fungi were found in the current study for the CDH cluster from Ascomycota to Basidiomycota. However, HGT has recently become somewhat of a default explanation for all genes that do not fit the expected evolutionary models [23]. We do not want to suggest HGT as the definitive answer here, but rather point out that multiple evolutionary models are possible for certain enzymes.

## Conclusions

This work offers new insights into the sequence variation and phylogenetic relationships of fungal GMC sequences, and thus should enable and support more detailed studies and annotations of putative GMC oxidoreductases. To make use of the full currently available amount of sequencing data, which exceeds the scale and diversity to be handled directly by phylogenetic methods, we used SSNs as a preparatory tool to cluster and functionally annotate selected sequences prior to a subsequent, more detailed evolutionary analysis. This approach allows for an unprecedented scale of sequence analysis for fungal GMC oxidoreductases. The overview of characterised and uncharacterised sequence space obtained by this work can be used as a basis for the discovery of novel enzymatic functions and elucidating enzyme specificities, that might be found for example in clades of enzymes activities identified by our phylogenetic analyses that are completely unexplored and uncharacterised to date. These novel clades we identified in these phylogenetic analyses are composed of hitherto uncharacterised sequences that can vary from known and studied sequences and one can expect that these will show properties and functionalities that distinguish them from known representatives of these enzyme subfamilies.

## Methods

### Generation of enzyme clusters

Starting from a selection of biochemically characterised fungal GMC members, we conducted two different database searches in November 2017. The first search was conducted with the HMMER tool [75] from EMBL-EBI (https://www.ebi.ac.uk/Tools/hmmer/), using profile hidden Markov Models to identify protein sequences in the UniProtKB database that display GMC domains. The input for the search was a Clustal Omega [83] alignment of these biochemically characterised sequences from literature as well as a single POx sequence (AAP40332) from *T. ochracea* because of a different pattern of Pfam domain hits for POx. The search was limited to the kingdom of fungi (taxon identifier: 4751) and hits were considered significant with an *E*-value ≤ 1.0^−35^. To select only for GMC oxidoreductases, search results were further restricted by their matches with Pfam domains and sequences containing any other major domain in addition to GMC_oxred_N (PF00732), GMC_oxred_C (PF05199), CDH-cyt (PF16010), or CBM (PF00734) were discarded.

The second search was conducted using BLAST on the NCBI database with two characterised sequences of each fungal GMC enzyme class, respectively (AOx from *Ogataea angusta*, CAA26278.1 and from *Phanerochaete chrysosporium*, CDG66232.1; AAO from *Pleurotus pulmonarius,* AAF31169.1 and from *Pycnoporus cinnabarinus*, ALS87661.1; CDH from *Crassicarpon hotsonii* (*Myriococcum thermophilum*), ABS45567.2 and from *Trametes cinnabarina,* ADX41688.1; GDH from *Aspergillus flavus*, XP002372599.1 and from *Pycnoporus cinnabarinus*, AIL89873.1; GOx from *Aspergillus niger*, AGI04246.1 and from *Talaromyces variabilis*, CAE47418.1; PDH from *Leucoagaricus meleagris*, 4H7U AAW82997.1 and from *Agaricus xanthodermus*, AHA85314.1; POx from *Trametes ochracea*, AAP40332.1 and from *Tricholoma matsutake*, Q8J2V8.1). The search was restricted to fungi (taxon identifier: 4751) and only sequences showing an identity of 35%–99% were selected.

A set of 99 annotated sequences from previous phylogenetic studies on GMC enzymes was added to help define the clusters in the sequence similarity network. Sequences containing invalid protein characters (B, J, O, U, X, or Z) were removed and the remaining sequences were filtered for a minimum length of 450 amino acids. Sequence redundancy was removed using CD-HIT [58] with a sequence identity cut-off of 0.99. The final selected set included 9385 fungal GMC sequences (7429 UniProtKB, 1857 NCBI and 99 additional annotated sequences).

The SSN was calculated using the web tool of Enzyme Function Initiative-Enzyme Similarity Tool (EFI-EST) (https://efi.igb.illinois.edu/efi-est/) [32] and edited with Cytoscape [82]. Based on the plots ‘Number of Edges at Score’ and ‘Percent Identity vs Alignment Score’ after the initial calculation of the SSN, the alignment score cut-off was set to 10^−85^, corresponding to a sequence identity of ~ 35% in the network. The alignment score cut-off was then gradually altered from 10^−85^ to 10^−140^ in steps of 10^−5^, thereby continuously displaying only more specific edges.

Additionally added annotated sequences were removed again from the five selected clusters when showing sequence redundancy. The tool SeqScrub was used for uniformly renaming all sequences of a cluster and collecting their taxonomic information [29]. All sequences of an individual cluster were aligned with MAFFT v7.271 [48] using the FFT-NS-2 method. Sequences were further selected to show three properties. Firstly, the well-known FAD-binding motif GxGxxG, which is part of the Rossmann fold [24], or the two variations GxGxxA and GxGxxS thereof, had to be part of the sequence. Secondly, another well-conserved FAD-associated motif in GMC enzymes with the consensus hGGpp or hGGGpp, where h is a hydrophobic residue and p a polar residue (positions 100–104 in *An*GOx 1CF3, 97–101 in *Lm*PDH 4H7U, 90–95 in *Kp*AOx 5HSA, 314–318 in *Nc*CDH 4QI7, and 161–165 in *To*POx 1TT0) had to be present. And thirdly, a catalytic His/His or His/Asn pair typical for GMC oxidoreductases [45, 96, 103] was used as a selection criterion. Sorting resulted in five clusters named AAO–PDH, AOx, CDH, GOx–GDH, and POx with 476, 720, 677, 471 and 95 sequences, respectively. Fasta files of these sequence selections are available as Additional files 8, 9, 10, 11 and 12.

### Generation of phylogenetic trees

The five sorted sequence clusters were again aligned individually by MAFFT v7.271 FFT-NS-2 [48] and alignments were trimmed for positions with > 99% gaps (> 95% for POx because of the small size of the cluster) by trimAl v1.2 [11]. Uninformative sites were removed from the alignment using Gblocks 0.91b [94] with a less stringent block selection, allowing for less strict flanking positions, setting minimum length of a block to five and allowed gap positions to “with half”. The alignment of the multidomain enzyme CDH was cut N-terminally six positions upstream of the conserved GxGxxG motif and C-terminally 18 positions downstream of the catalytic Asn, leaving only the GMC dehydrogenase domain. The optimal amino acid substitution model for each alignment was determined using ProtTest v3.4.2 [19] under the AIC criterion, resulting in LG [54] for all alignments (using the BIC criterion resulted in the same optimal model). Phylogenetic trees were calculated by PhyML on the Montpellier Bioinformatics Platform (http://www.atgc-montpellier.fr/phyml/) [35] using default settings with SPR moves to optimise tree topology and aLRT SH-like branch support. All trees were rooted on midpoint and visualised in MEGA7 [53]. Newick files of the midpoint-rooted trees are available as Additional files 13, 14, 15, 16 and 17. Clades in the respective trees were defined individually based primarily on topology, on taxonomy and when necessary also on the characterised sequence space in a final step.

Based on the trimAl v1.2 trimmed alignment of the CDH cluster, a separate selection for functional cytochrome domains was created by cutting off all dehydrogenase domains including the linker sequence. A cytochrome domain was considered functional if it showed the two axial heme-ligating residues Met and His and two Cys residues forming a disulfide bridge as described (M65, H163, C121, and C124 in *Pc*Cyt-CDH, 1D7B; [38]). All sequences were named according to the CDH clade to which they belonged in the dehydrogenase domain tree (CDH I, CDH II, or CDH III), then re-aligned by MAFFT v7.271, with uninformative sites removed from the alignment using Gblocks 0.91b with less stringent criteria as described above. Phylogeny was assessed using PhyML with default settings, SPR moves, aLRT SH-like branch support, and the Smart Model Selection [56]. The inferred tree was rooted on midpoint and visualised in MEGA7 [53]. Species trees were downloaded from http://www.timetree.org/ [52] showing the most common order of fungi.

### Sequence analysis

N-terminal signal sequences were predicted using the SignalP 4.1 server [69] (http://www.cbs.dtu.dk/services/SignalP/) with default settings for eukaryotes. Sequence logos were created on https://weblogo.berkeley.edu/logo.cgi. The fractions of different domains present in CDH clades were determined using the hmmscan function of the HMMER tool [75] from EMBL-EBI (https://www.ebi.ac.uk/Tools/hmmer/search/hmmscan) searching the Pfam database. Mean percent sequence identity was calculated from the number of identical positions for every pair of sequences taken from the sorted cluster alignment without realigning. Positions where one or both sequences had a gap were not considered in the calculations. Exon counts were retrieved from the associated NCBI or EnsembelGenome record for each sequence. Sequences that mapped to mRNA records and sequences with no associated exon information were excluded (71 sequences in total). Alignment overviews were created in Jalview v2 [100] and amino acids were coloured according to the Zappo colour scheme. Visualisation of AOx crystal structure (PDB 5HSA) was done in PyMOL 2.0.7 (The PyMOL Molecular Graphics System, Version 2.0.7 Schrödinger, LLC).

## Additional files

**Additional file 1: Figure S1A.** Sequence similarity network at an alignment score cut-off of 10^−105^. **B.** Sequence similarity network at an alignment score cut-off of 10^−135^.

**Additional file 2: Figure S2A.** Taxonomic distribution of fungal AAO–PDH. **B.** Taxonomic distribution of fungal AOx. **C.** Taxonomic distribution of fungal CDH. **D.** Taxonomic distribution of fungal GOx-GDH. **E.** Taxonomic distribution of fungal POx.

**Additional file 3: Figure S3A.** Maximum likelihood tree of AAO–PDH with the corresponding alignment overview. **B.** Maximum likelihood tree of AOx with the corresponding alignment overview. **C.** Maximum likelihood tree of CDH with the corresponding alignment overview. **D.** Maximum likelihood tree of GOx-GDH with the corresponding alignment overview. **E.** Maximum likelihood tree of POx with the corresponding alignment overview.

**Additional file 4: Figure S4.** Sequence logos for comparison of the active site architecture in the three clades of the AAO–PDH cluster, AAO, AAO-like, and PDH.

**Additional file 5: Figure S5.** Crystal structure of *Komagataella phaffii* AOx (5HSA) showing the C-terminal extension (**A**) and the insertion (**B**) when compared to AOx-like sequences.

**Additional file 6: Figure S6.** Sequence logos of the ten last amino acids of all sequences in each clade of the AOx cluster.

**Additional file 7: Figure S7.** Maximum likelihood tree of all cytochrome domains present in the CDH cluster.

**Additional file 8.** Fasta file of all sequences from the AAO–PDH cluster.

**Additional file 9.** Fasta file of all sequences from the AOx cluster.

**Additional file 10.** Fasta file of all sequences from the CDH cluster.

**Additional file 11.** Fasta file of all sequences from the GOx-GDH cluster.

**Additional file 12.** Fasta file of all sequences from the POx cluster.

**Additional file 13.** Newick file of maximum likelihood tree of the AAO–PDH cluster.

**Additional file 14.** Newick file of maximum likelihood tree of the AOx cluster.

**Additional file 15.** Newick file of maximum likelihood tree of the CDH cluster.

**Additional file 16.** Newick file of maximum likelihood tree of the GOx-GDH cluster.

**Additional file 17.** Newick file of maximum likelihood tree of the POx cluster.

## Data Availability

The datasets used and/or analysed during the current study are available from the corresponding author on reasonable request.

## Abbreviations

AADH: aryl-alcohol dehydrogenase
AAO: aryl-alcohol oxidoreductase
AAOx: aryl-alcohol oxidase
AOx: alcohol oxidase
AS: alignment score
CBM: carbohydrate-binding module
CDH: cellobiose dehydrogenase
FAD: flavin adenine dinucleotide
GDH: glucose dehydrogenase
GMC: glucose–methanol–choline
GOx: glucose oxidase
HGT: horizontal gene transfer
MSA: multiple sequence alignment
PDH: pyranose dehydrogenase
POx: pyranose oxidase
PTS: peroxisomal targeting signal
SSN: sequence similarity network
