# Supplementary material for: The GMC superfamily of oxidoreductases revisited: analysis and evolution of fungal GMC oxidoreductases
Source: Biotechnol Biofuels. 2019 May 10;12:118. doi: 10.1186/s13068-019-1457-0 (PMC6509819; doi:10.1186/s13068-019-1457-0)
Supplement: Supplementary file 1 — Additional file 1: Figure S1A. Sequence similarity network at an alignment score cut-off of 10−105. B. Sequence similarity network at an alignment score cut-off of 10−135. [file 13068_2019_1457_MOESM1_ESM.docx]

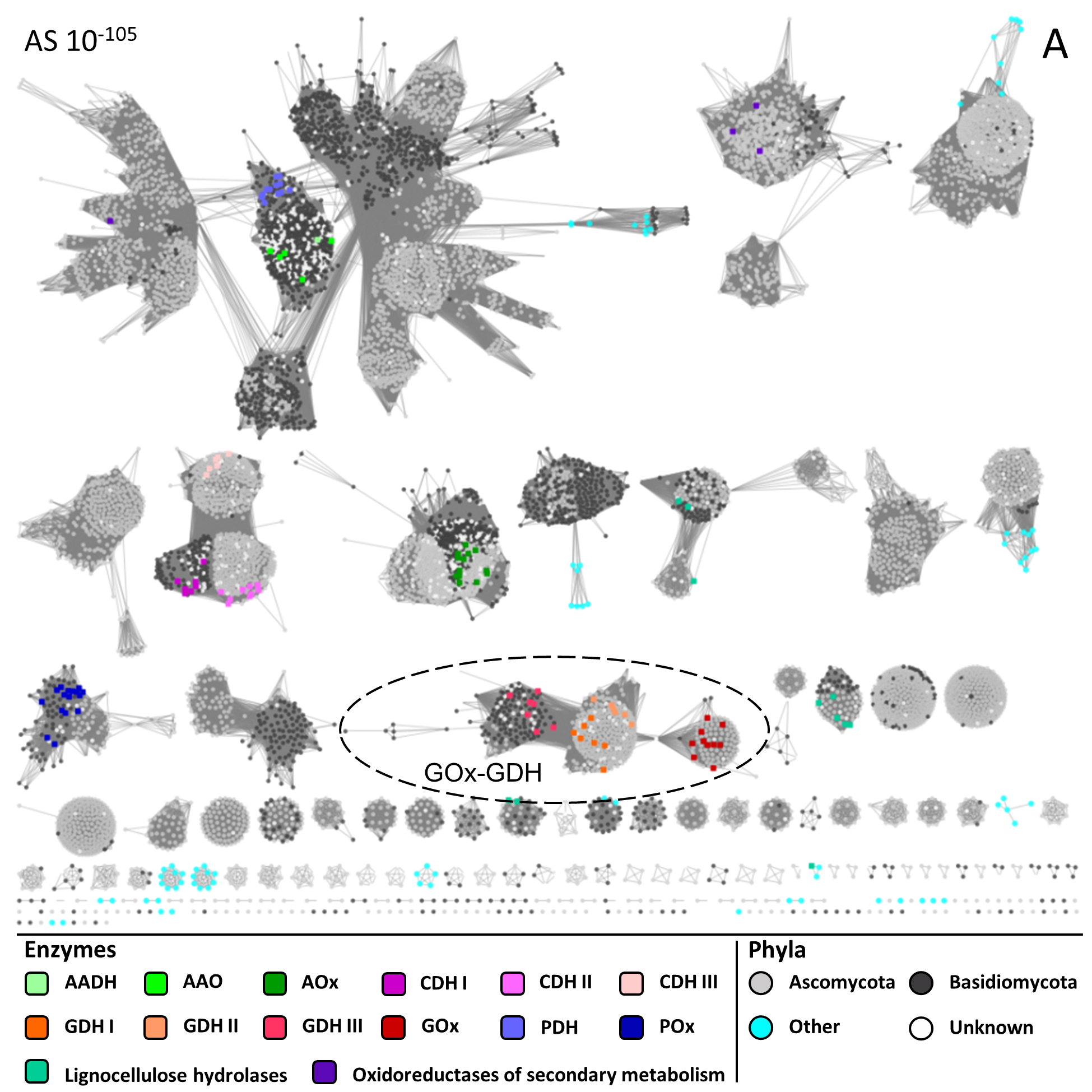


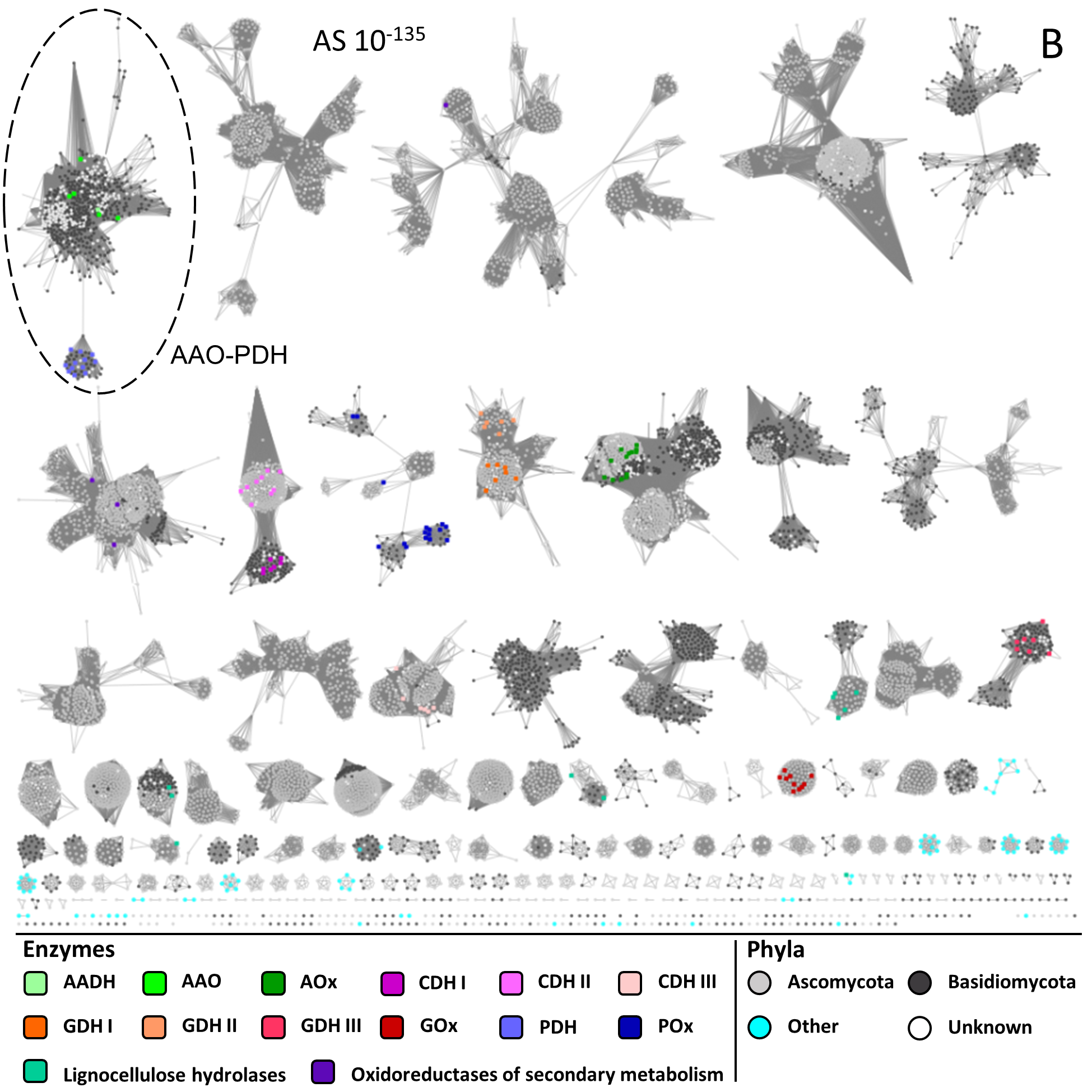


**Figure S1. A: Sequence similarity network at an alignment score cut-off of 10^-105^. B: Sequence similarity network at an alignment score cut-off of 10^-135^.**Clusters extracted at this cut-off are indicated by dashed circles. Annotated sequences are coloured according to their functionality ('Enzymes'). All other sequences are coloured according to the fungal phyla they occur in ('Phyla').
