# Supplementary material for: The GMC superfamily of oxidoreductases revisited: analysis and evolution of fungal GMC oxidoreductases
Source: Biotechnol Biofuels. 2019 May 10;12:118. doi: 10.1186/s13068-019-1457-0 (PMC6509819; doi:10.1186/s13068-019-1457-0)
Supplement: Supplementary file 2 — Additional file 2: Figure S2A. Taxonomic distribution of fungal AAO–PDH. B. Taxonomic distribution of fungal AOx. C. Taxonomic distribution of fungal CDH. D. Taxonomic distribution of fungal GOx-GDH. E. Taxonomic distribution of fungal POx. [file 13068_2019_1457_MOESM2_ESM.docx]

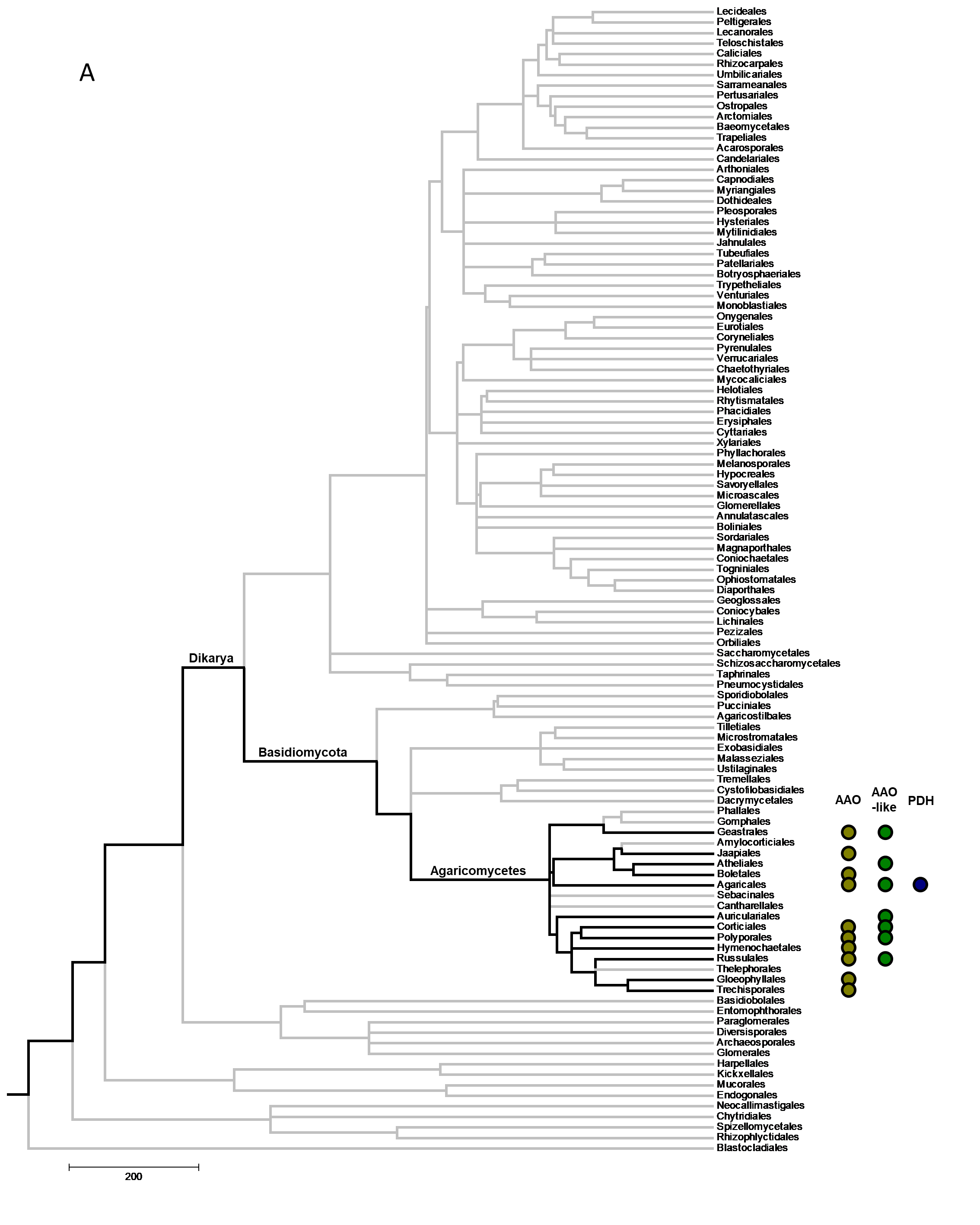


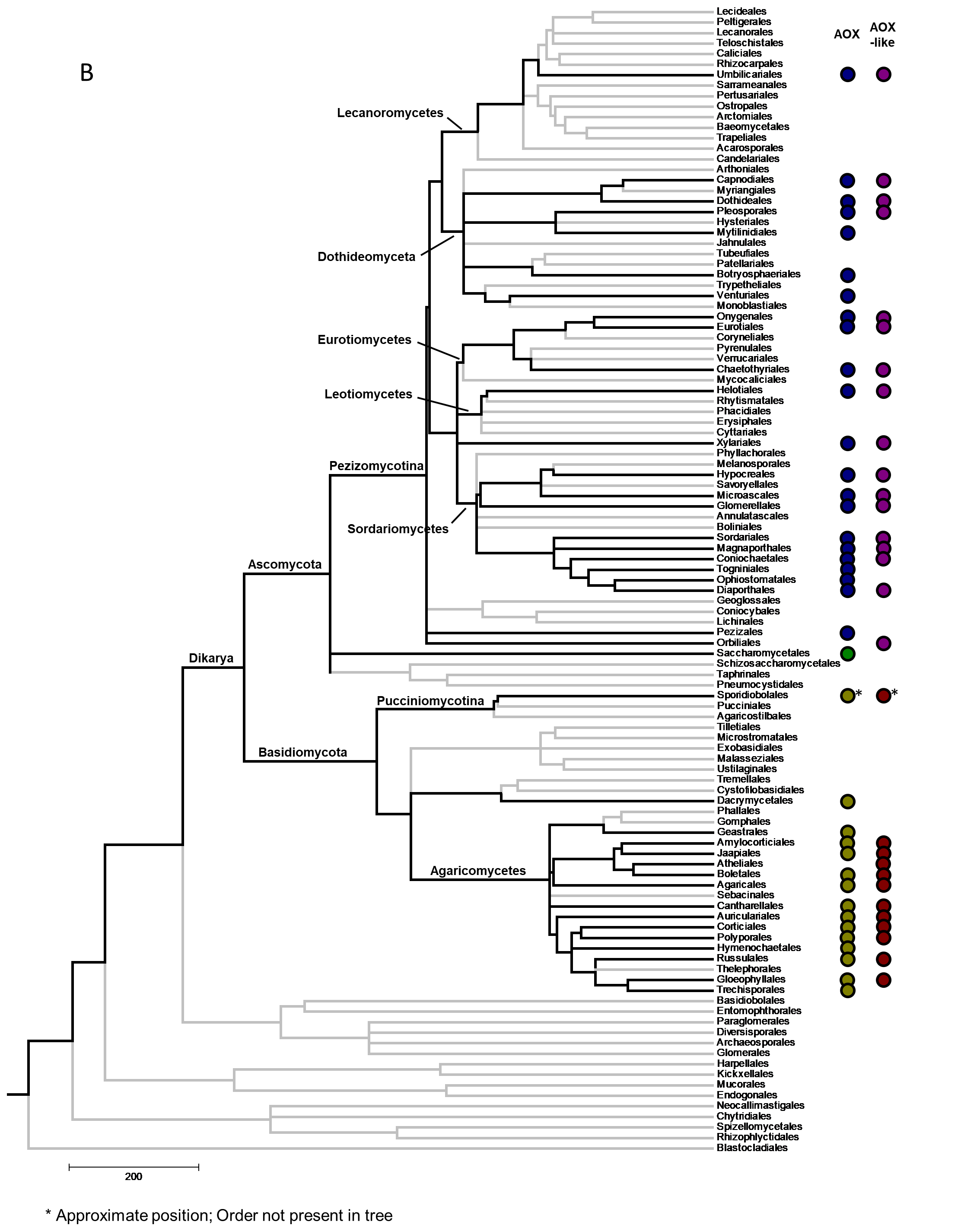


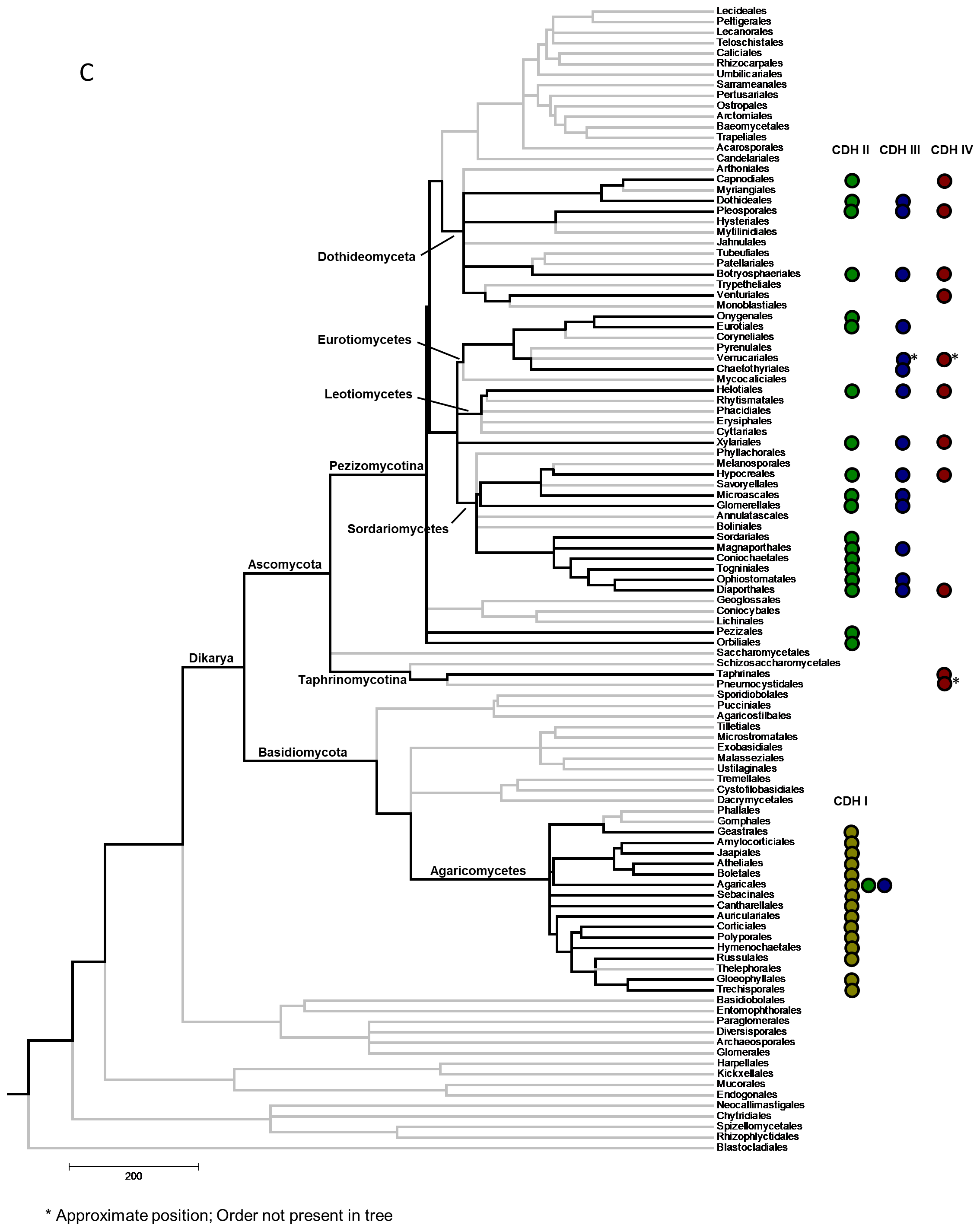


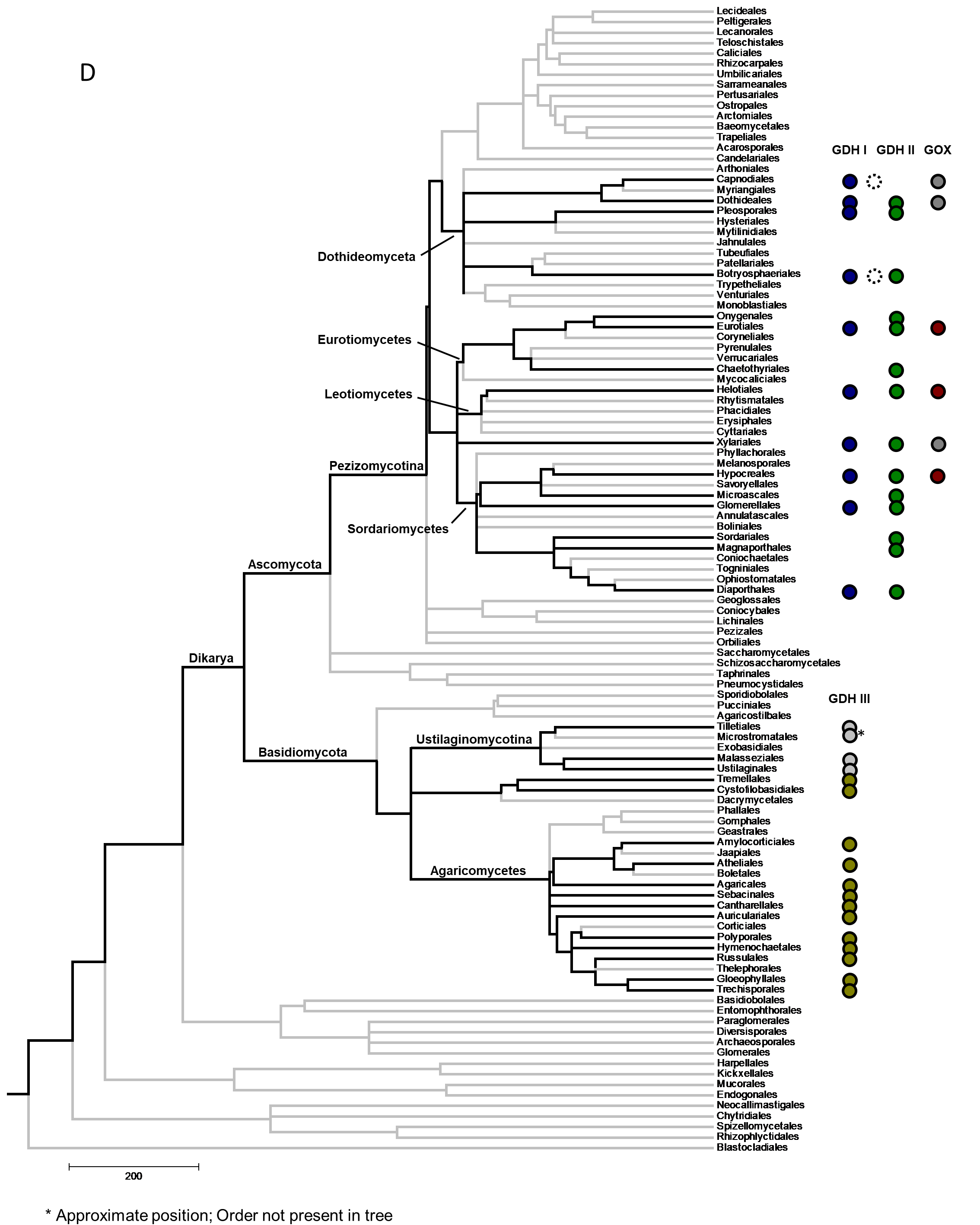


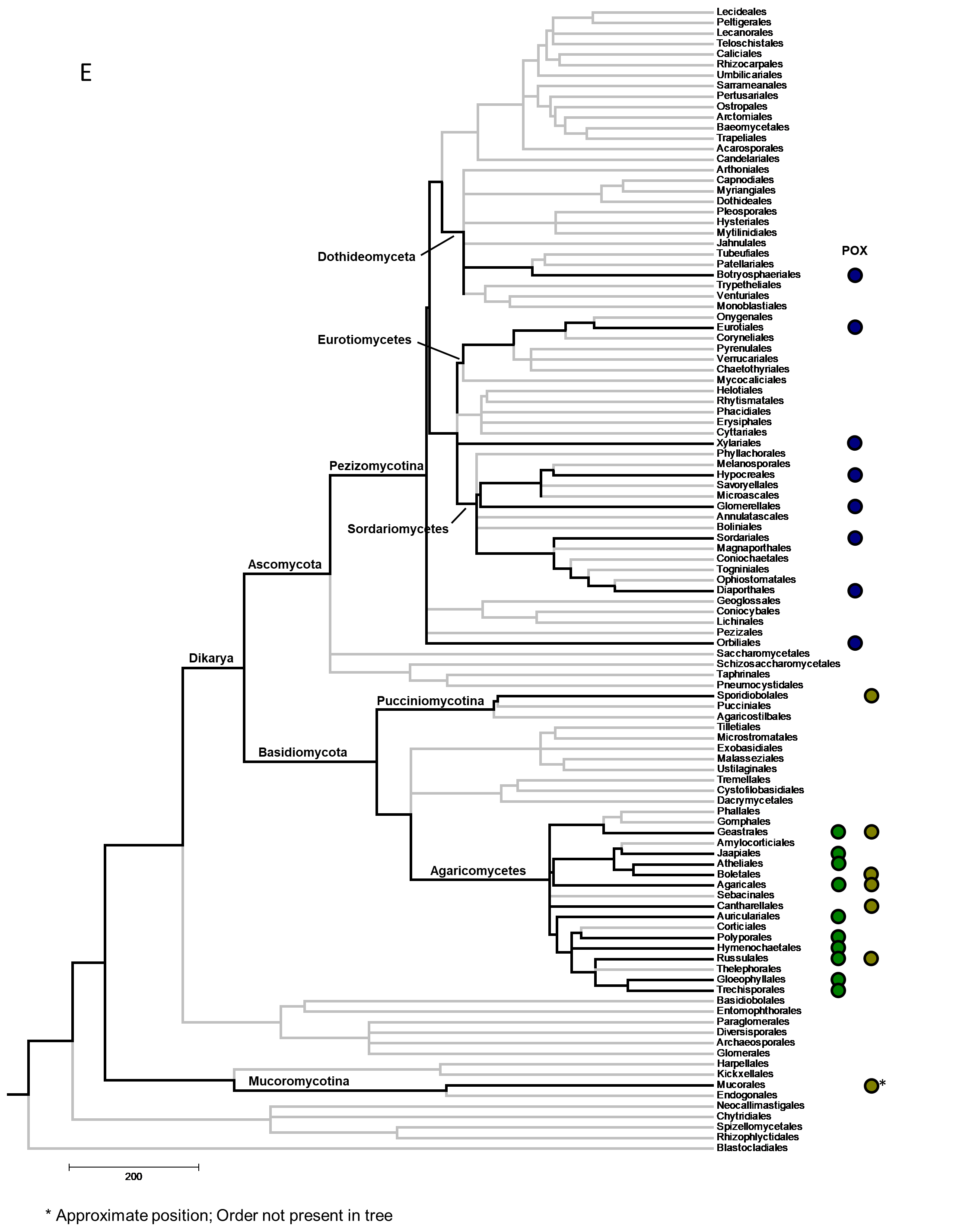


Figure S2. Taxonomic distribution of fungal GMC / AA3 sequences.

Species tree showing fungal orders was downloaded from Timetree.org. Coloured circles and thick lines mark fungal orders that are represented by at least one sequence in the following clusters: **A**, AAO-PDH cluster (colour coding as in Figure 2); **B**, AOx cluster (colour coding as in Figure 3); **C**, CDH cluster (colour coding as in Figure 4); **D**, GOx-GDH cluster (colour coding as in Figure 5); **E**, POx cluster (colour coding as in Figure 6).
