# Supplementary material for: The GMC superfamily of oxidoreductases revisited: analysis and evolution of fungal GMC oxidoreductases
Source: Biotechnol Biofuels. 2019 May 10;12:118. doi: 10.1186/s13068-019-1457-0 (PMC6509819; doi:10.1186/s13068-019-1457-0)
Supplement: Supplementary file 3 — Additional file 3: Figure S3A. Maximum likelihood tree of AAO–PDH with the corresponding alignment overview. B. Maximum likelihood tree of AOx with the corresponding alignment overview. C. Maximum likelihood tree of CDH with the corresponding alignment overview. D. Maximum likelihood tree of GOx-GDH with the corresponding alignment overview. E. Maximum likelihood tree of POx with the corresponding alignment overview. [file 13068_2019_1457_MOESM3_ESM.docx]

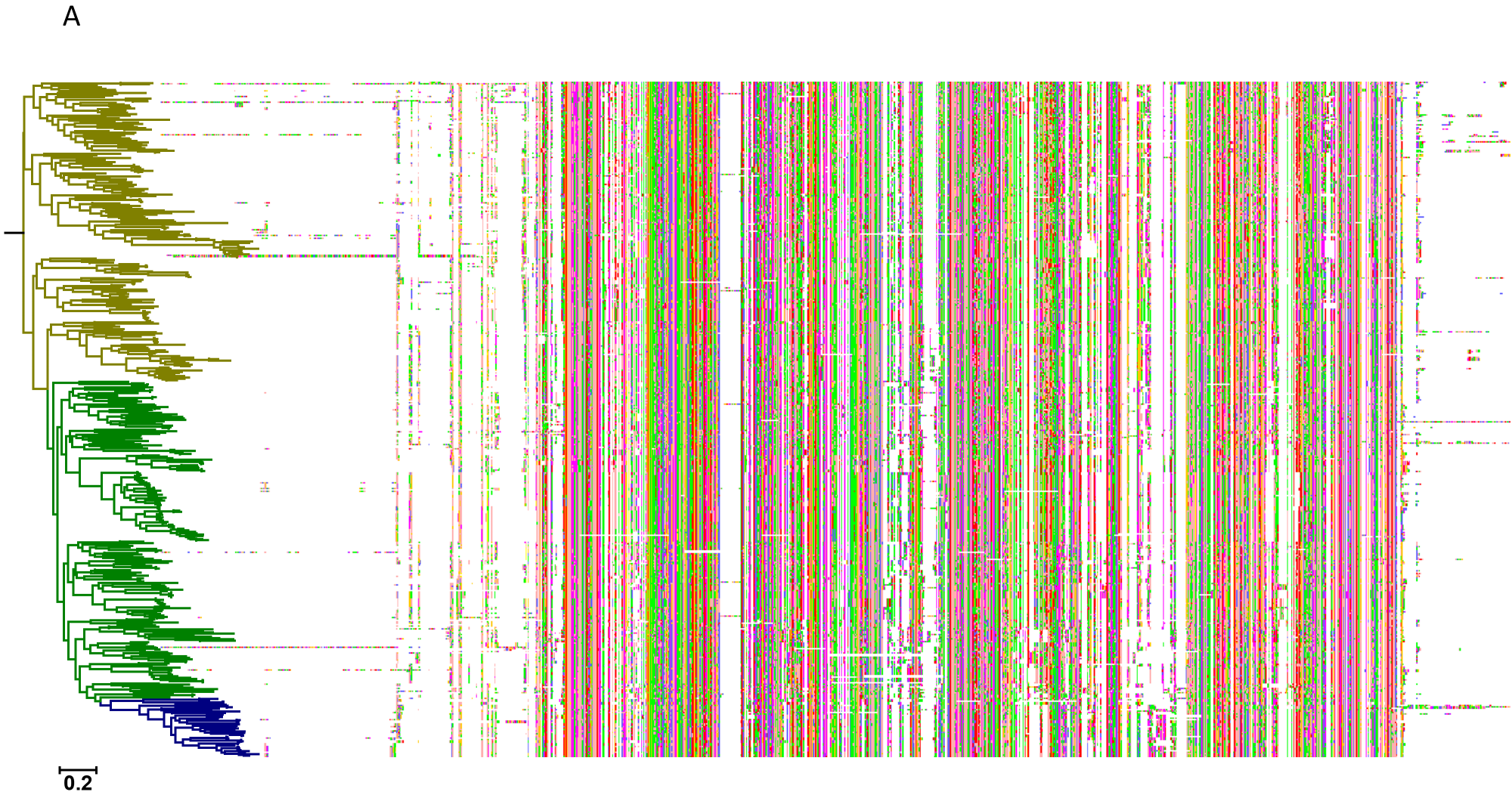


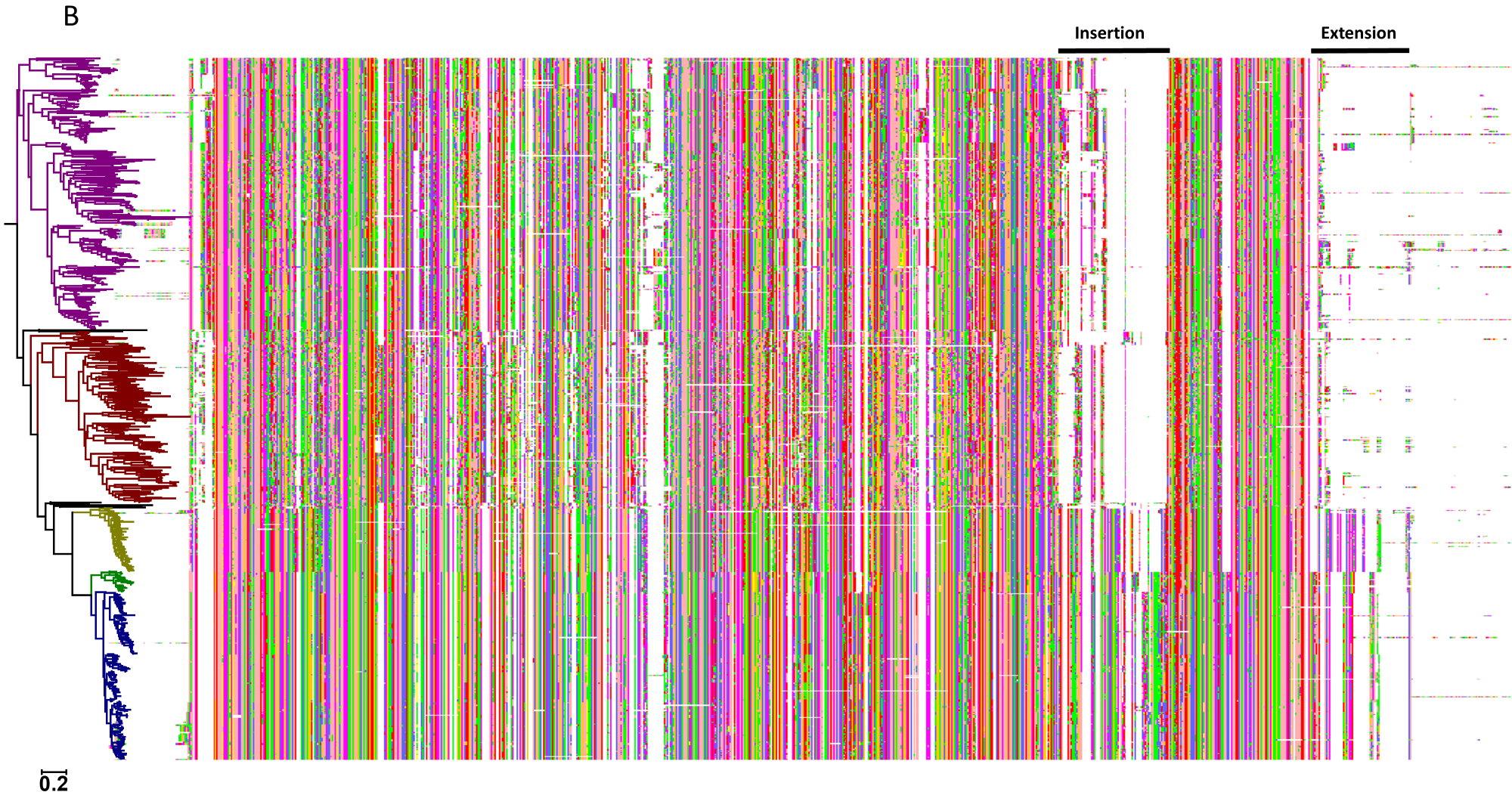


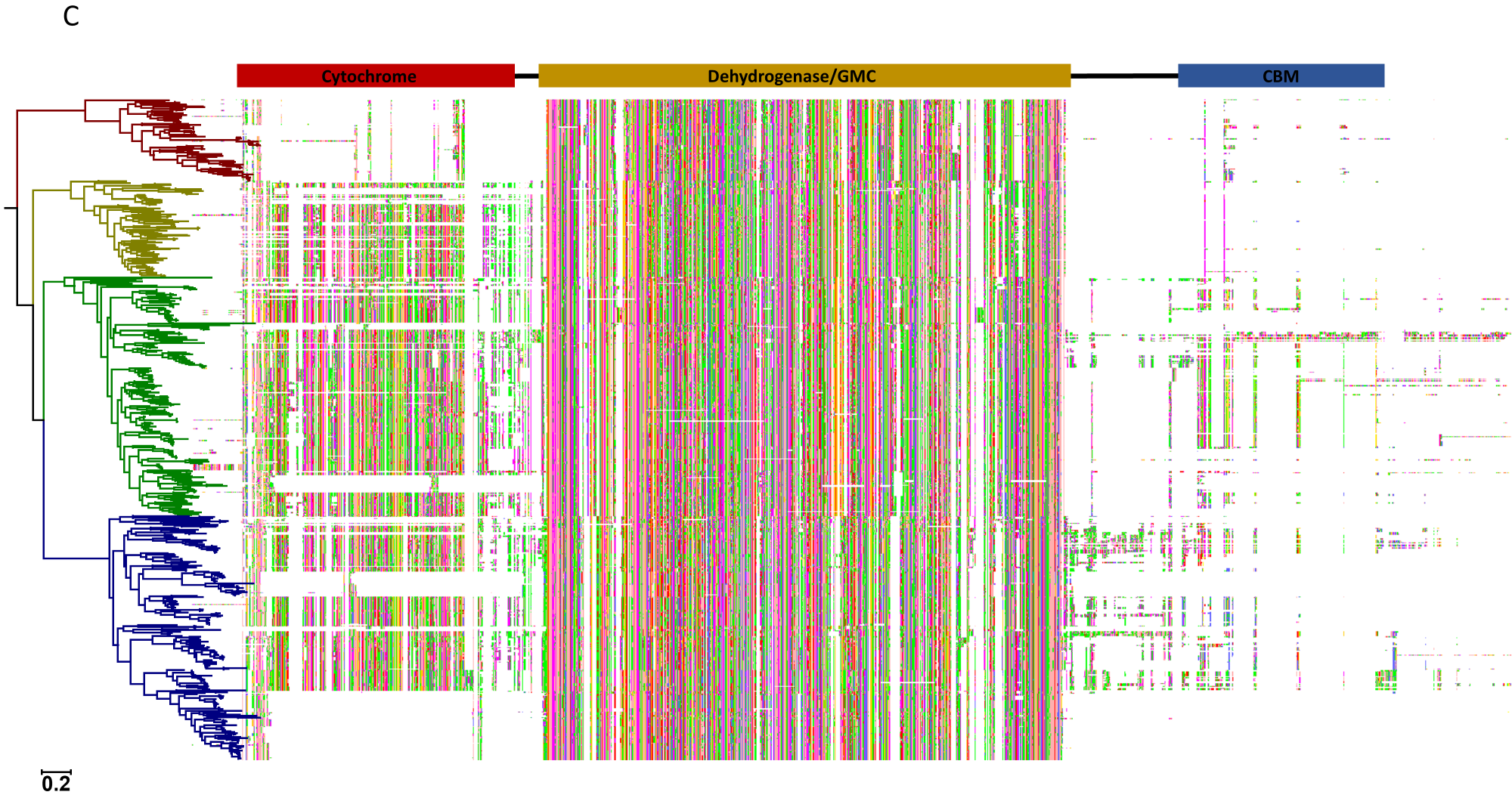


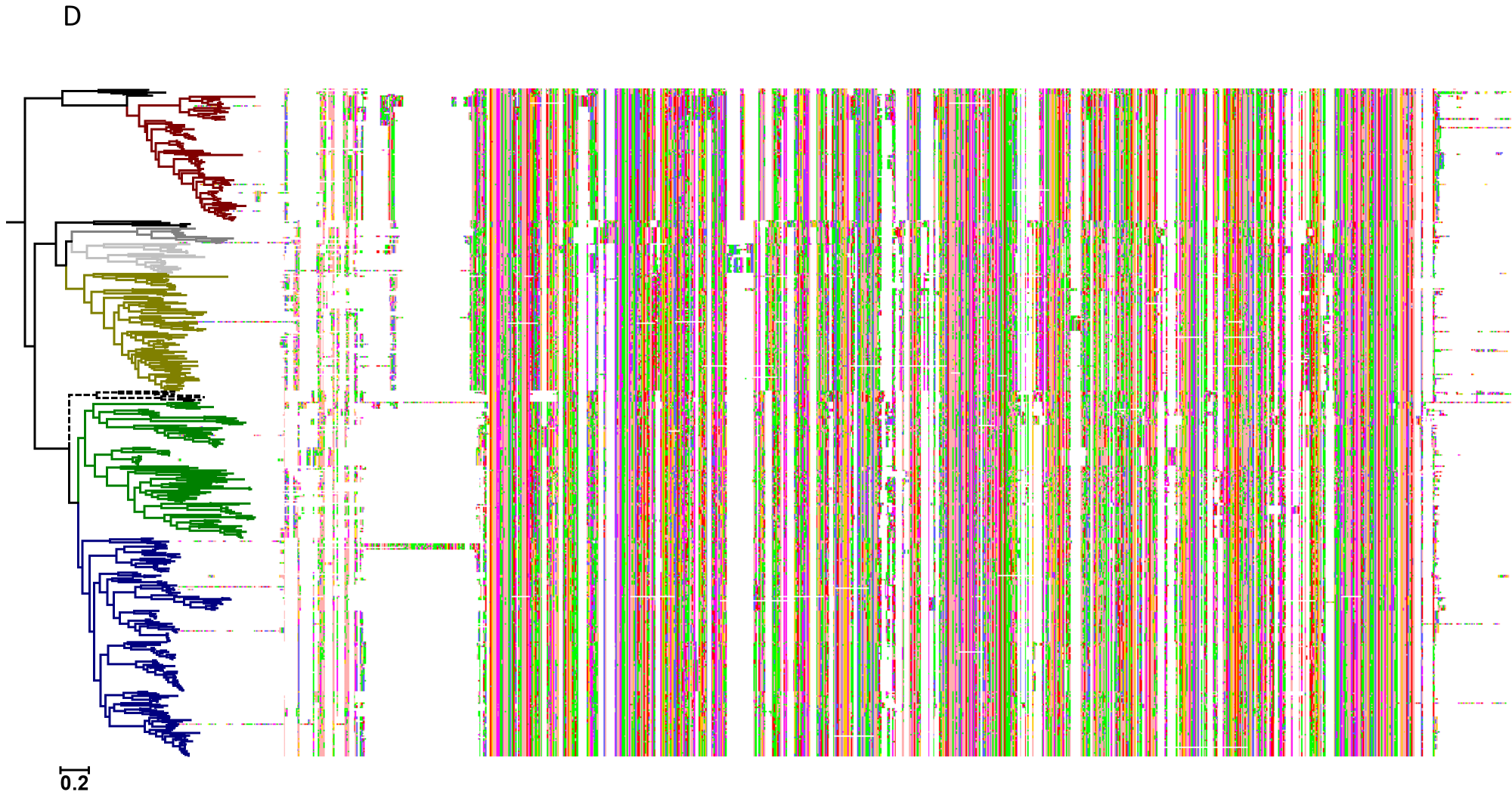


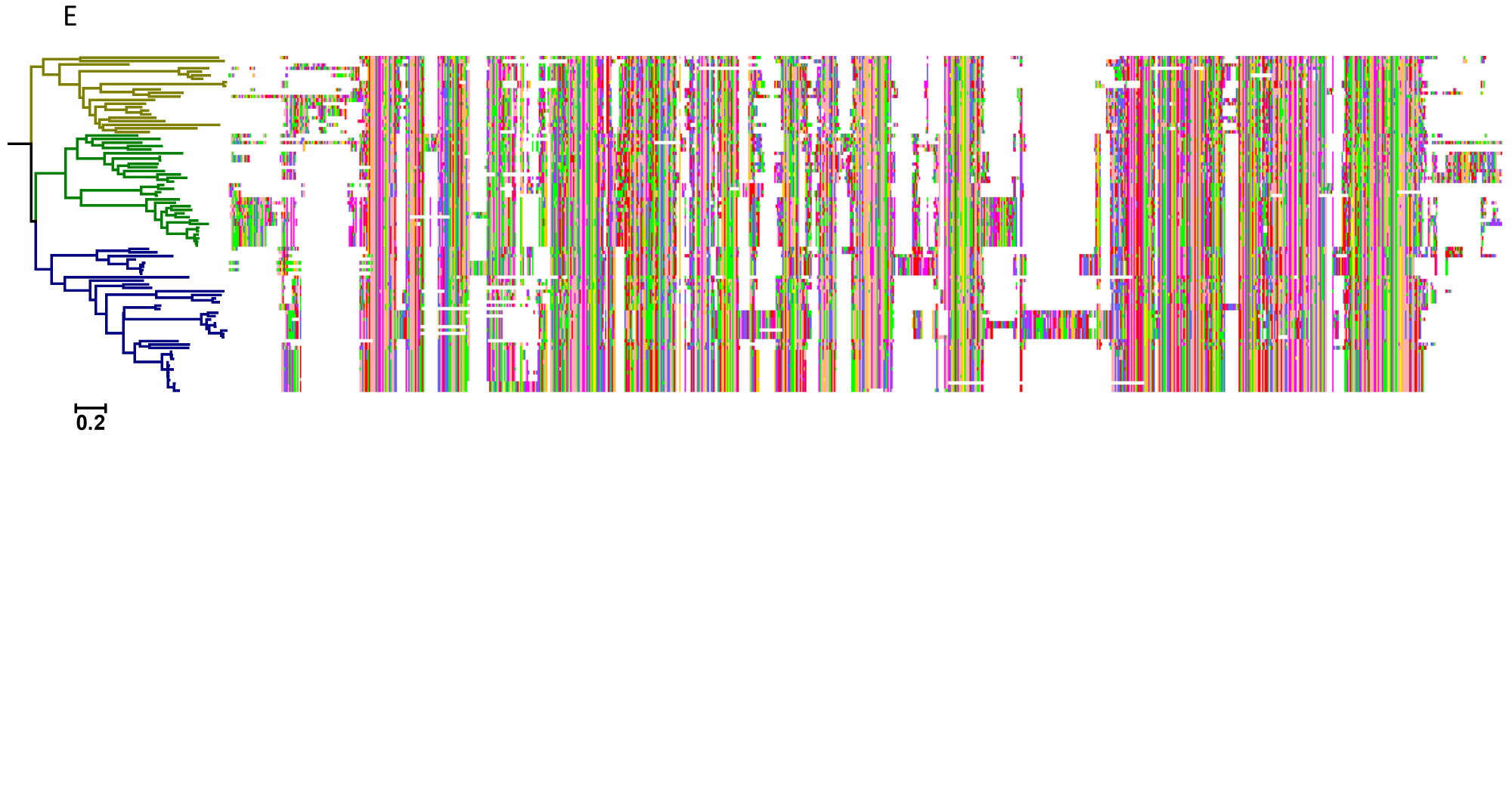


Figure S3. Maximum likelihood tree of the respective GMC oxidoreductases with their corresponding alignment overviews trimmed for positions with >99% gaps (>95% in case of POx).

**A**, AAO-PDH; **B**, AOx; **C**, CHD; **D**, GOx-GDH; **E**, POx.
