# Supplementary material for: The GMC superfamily of oxidoreductases revisited: analysis and evolution of fungal GMC oxidoreductases
Source: Biotechnol Biofuels. 2019 May 10;12:118. doi: 10.1186/s13068-019-1457-0 (PMC6509819; doi:10.1186/s13068-019-1457-0)
Supplement: Supplementary file 4 — Additional file 4: Figure S4. Sequence logos for comparison of the active site architecture in the three clades of the AAO–PDH cluster, AAO, AAO-like, and PDH. [file 13068_2019_1457_MOESM4_ESM.docx]

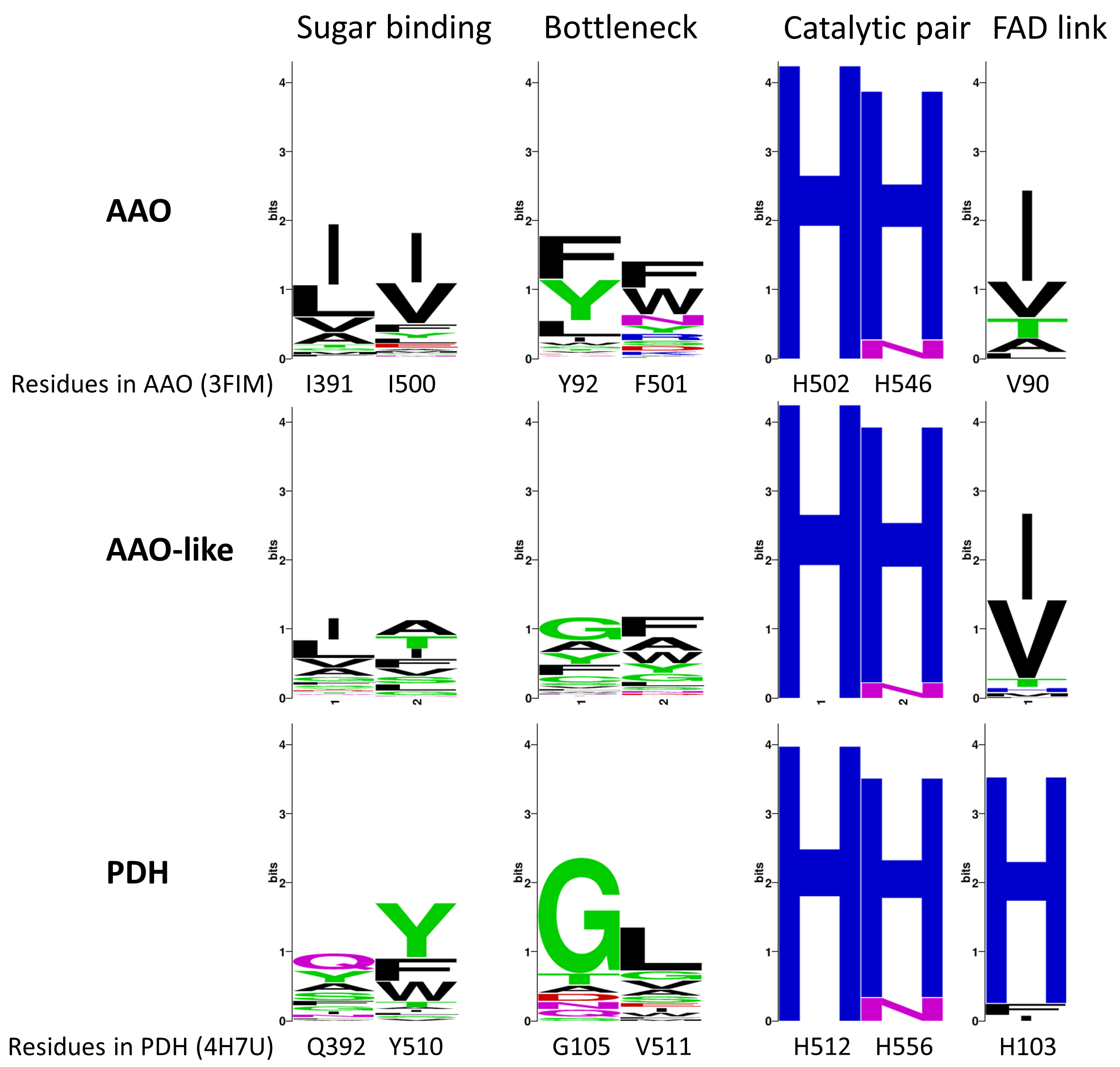


Figure S4. Sequence logos for comparison of the active site architecture in the three clades of the AAO-PDH cluster, AAO, AAO-like, and PDH. The shown residues are responsible for substrate binding ('Sugar binding' in PDH), active site accessibility ('Bottleneck' in AAO), substrate conversion ('Catalytic pair'), and forming a covalent linkage with FAD ('FAD link' in PDH).
