# Supplementary material for: The GMC superfamily of oxidoreductases revisited: analysis and evolution of fungal GMC oxidoreductases
Source: Biotechnol Biofuels. 2019 May 10;12:118. doi: 10.1186/s13068-019-1457-0 (PMC6509819; doi:10.1186/s13068-019-1457-0)
Supplement: Supplementary file 5 — Additional file 5: Figure S5. Crystal structure of Komagataella phaffii AOx (5HSA) showing the C-terminal extension (A) and the insertion (B) when compared to AOx-like sequences. [file 13068_2019_1457_MOESM5_ESM.docx]

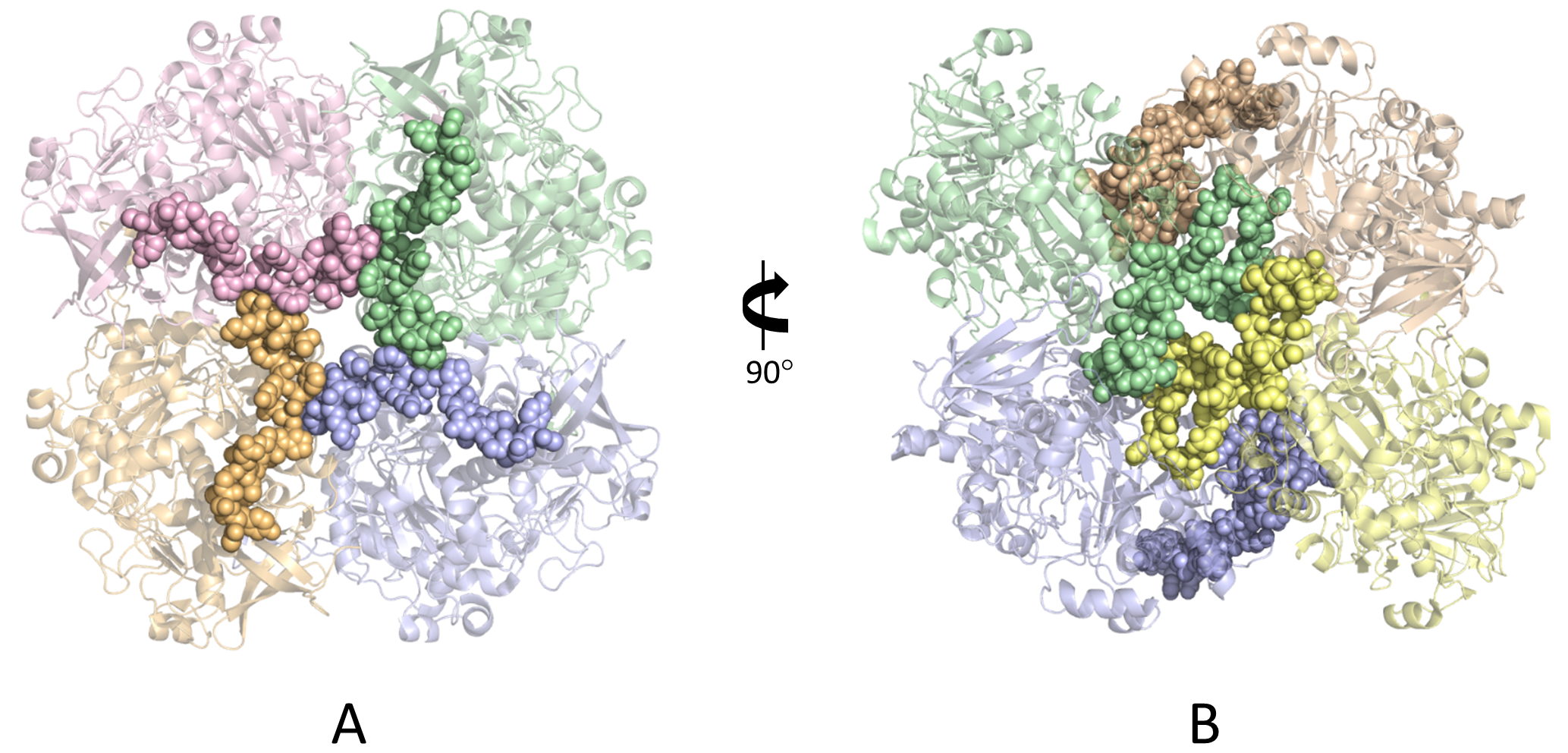


Figure S5. Crystal structure of *Komagataella phaffii* AOx (5HSA). This figure shows the C-terminal extension involved in the formation of the tetramer (A) and the insertion interacting with two other subunits from both tetramers to support octamer formation (B). Both regions are displayed as spheres.
