# Supplementary material for: The GMC superfamily of oxidoreductases revisited: analysis and evolution of fungal GMC oxidoreductases
Source: Biotechnol Biofuels. 2019 May 10;12:118. doi: 10.1186/s13068-019-1457-0 (PMC6509819; doi:10.1186/s13068-019-1457-0)
Supplement: Supplementary file 6 — Additional file 6: Figure S6. Sequence logos of the ten last amino acids of all sequences in each clade of the AOx cluster. [file 13068_2019_1457_MOESM6_ESM.docx]

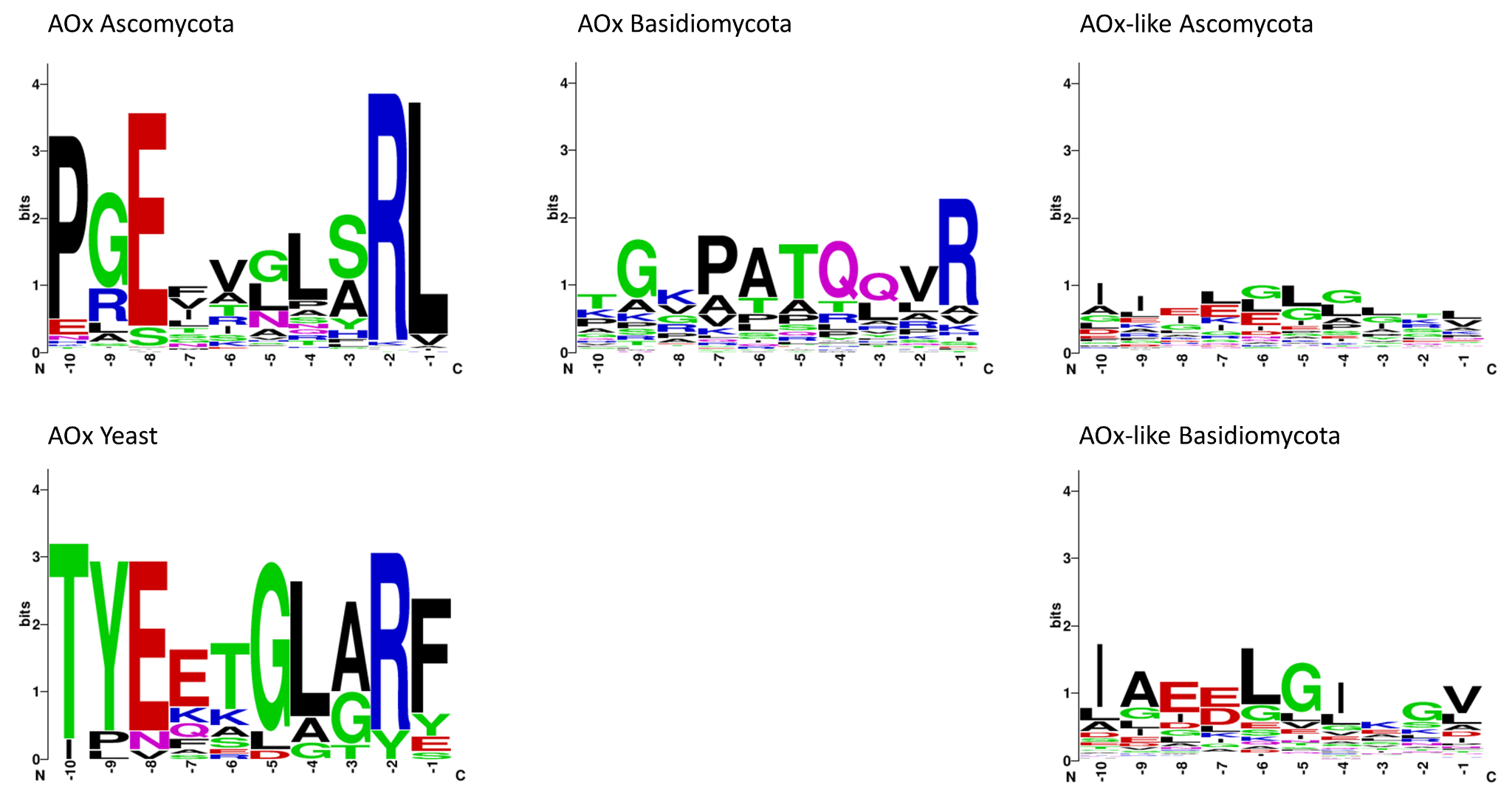


**Figure S6. Sequence logos of the ten last amino acids of all sequences in each clade of the AOx cluster.** Only Ascomycota (including yeast) sequences show a conserved sequence motif matching the PTS motif known from literature (Nötzel et al. 2016).

Nötzel C, Lingner T, Klingenberg H, Thoms S. 2016. Identification of new fungal peroxisomal matrix proteins and revision of the PTS1 consensus. *Traffic* 17:1110-1124.
