# Supplementary material for: The GMC superfamily of oxidoreductases revisited: analysis and evolution of fungal GMC oxidoreductases
Source: Biotechnol Biofuels. 2019 May 10;12:118. doi: 10.1186/s13068-019-1457-0 (PMC6509819; doi:10.1186/s13068-019-1457-0)
Supplement: Supplementary file 7 — Additional file 7: Figure S7. Maximum likelihood tree of all cytochrome domains present in the CDH cluster. [file 13068_2019_1457_MOESM7_ESM.docx]

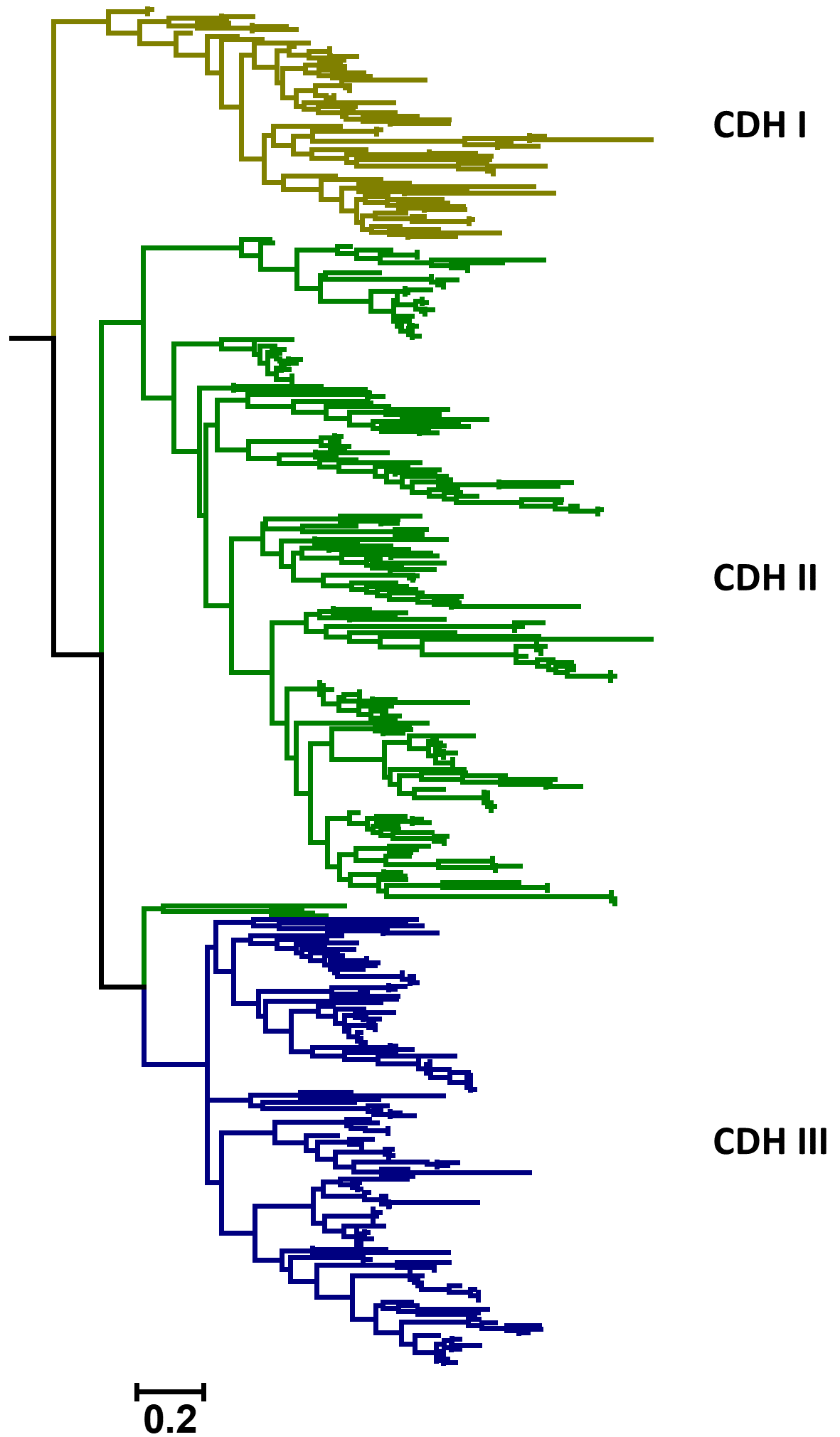


**Figure S7. Maximum likelihood tree of all cytochrome domains present in the CDH cluster.** The colouring of the individual branches was kept the same as defined in the clades from the tree based on dehydrogenase domains only (Figure 4 and Figure S3C). The general topology of the cytochrome domain tree is in accordance with the topology found for the dehydrogenase domains.
